# Molecular Insights into the Incorporation of Platinum-Based Drugs into Lipid Aggregates

**DOI:** 10.1021/acsomega.5c11203

**Published:** 2026-02-22

**Authors:** Kacper Rzepiela, Yousef Najajreh, Aneta Buczek, Birgit Strodel, Hebah Fatafta

**Affiliations:** † Faculty of Chemistry and Pharmacy, 49576 University of Opole, Oleska 48, 45-052 Opole, Poland; ‡ Forschungszentrum Jülich, Institute of Biological Information Processing: Structural Biochemistry (IBI-7), Wilhelm-Johnen-Str., 52428 Jülich, Germany; § Faculty of Pharmacy, 61147 Al-Quds University, Abu-Dies, P.O. Box 20002 Jerusalem, Palestine; ∥ Faculty of Production Engineering and Logistics, Opole University of Technology, Mikołajczyka 5, 45-271 Opole, Poland; ⊥ Faculty of Mathematics and Natural Sciences, Heinrich Heine University Düsseldorf, Universitätsstr. 1, 40225 Düsseldorf, Germany; # Department of Engineering and Communication, 52784 Bonn-Rhein-Sieg University of Applied Sciences, Grantham-Allee 20, 53757 Sankt Augustin, Germany

## Abstract

Platinum-based (Pt-based) compounds remain a cornerstone of chemotherapy, yet their clinical use is limited mainly due to poor tumor specificity and systemic toxicities. Fatty acid conjugation has emerged as a promising strategy to overcome the limitations of conventional platinum drugs by enhancing lipophilicity, improving cellular uptake, and potentially acting as prodrugs with altered physicochemical properties and binding kinetics to biomolecular targets. The covalent conjugation of lipophilic fatty acids also improves the compatibility of Pt-based compounds with lipid-based delivery systems, facilitating their incorporation. In this study, we employed atomistic molecular dynamics (MD) simulations to investigate the interactions between a series of Pt-based compounds, including cisplatin and fatty acid–conjugated analogs (CapryP, ArP, SteariP, ElaidP, and OleP), and biologically relevant phospholipids (DOPC, DSPE, and DPPG). Simulations revealed the spontaneous self-assembly of lipid–drug mixtures into micelle-like aggregates, driven by hydrophobic interactions and modulated by the chemical structure of the conjugated moieties. Cluster analysis demonstrated variation in aggregation dynamics among the compounds, with hydrophobic chain length and unsaturation degree influencing the rate and stability of complex formation. These findings provide insights at the molecular-level, shedding light into the molecular attributes that govern the incorporation of fatty acid–Pt-based conjugates into lipid assemblies, highlighting the potential of structural modifications to enhance their delivery within lipid-based systems.

## Graphical abstract

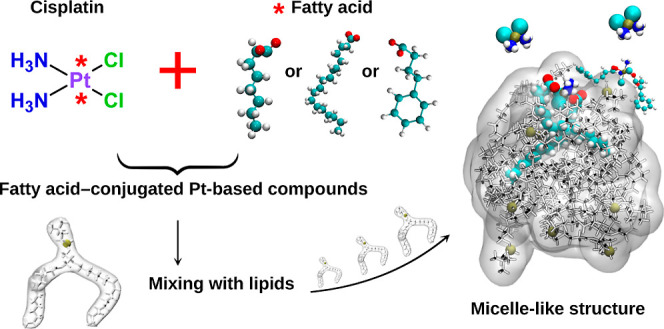

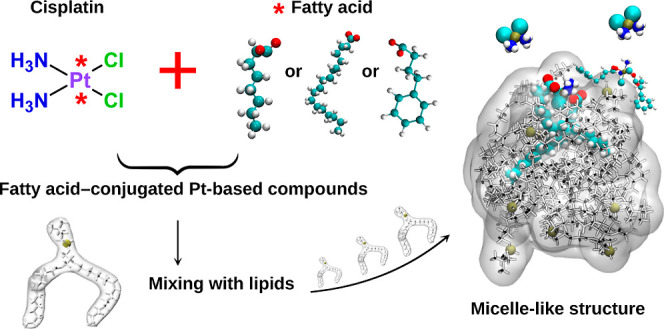

## Introduction

Chemotherapy is a widely adopted approach to cancer treatment. Platinum-based (Pt-based) compounds play a central role due to their potent cytotoxic effects. Cisplatin, the first Pt-based clinically administered drug, was discovered in the late 1960s^1^ and granted the FDA-approval in 1978, initially for treatment of testicular cancer. In a latter stage, the drug was approved for other types such as advanced ovarian and advanced bladder cancers. The drug is widely used in combination chemotherapy for a broader range of solid tumors including on-small cell lung cancer (NSCLC) and small cell lung cancer (SCLC), head and neck, esophageal, gastric, cervical, mesothelioma and breast cancer.^2^ Despite its effectiveness, cisplatin’s nonspecific mechanism of action often results in systemic toxicity,^3^ damaging healthy tissues, especially upon recurrent and prolonged administration.

To overcome these limitations, second- and third-generation Pt-based drugs such as carboplatin (1986) and oxaliplatin (1996) were developed. The main aims were to improve efficacy and reduce adverse effects associated with cisplatin, but also to overcome the inherent as well as the acquired resistance developed by cancer cells. The rapid ligand exchange reactions of platinum­(II)-based compounds are believed to be a major contributor to their severe toxicities. This understanding led to the adoption of kinetically inert platinum­(IV)-based complexes as a strategy to overcome these drawbacks. The key insight is that platinum­(IV) compounds can act as prodrugs, transforming into their more active platinum­(II) counterparts through a bioreductive process within the body. This mechanism aims to reduce off-target side effects by ensuring that the active drug is generated primarily where it is needed.^4^ Satraplatin, the most clinically advanced Pt­(IV) candidate, was designed for oral administration and has an improved toxicity profile. It was initially developed as a proof of concept to demonstrate the clinical viability of Pt­(IV) prodrug strategies. However, clinical failures, including a lack of regulatory approval,^5^ have raised concerns about the rationale of prodrugs and the reduction mechanism in the extracellular environment.

Two major challenges are rendering Pt-based chemotherapy from wide and safe indications: low tumor specificity and the emergence of resistance. These compounds indiscriminately target both cancerous and healthy cells, leading to adverse side effects such as bone marrow suppression and decreased blood cell counts. This high systemic toxicity limits the maximum safe dosage and ultimately reduces the overall antitumor efficacy of the treatment.^6^ Consequently, the quest for novel drug delivery strategies is intensifying, driven by the value of improving therapeutic effectiveness while simultaneously reducing harmful side effects.^7^

One promising strategy for enhancing the therapeutic potential of Pt-based compounds is the covalent conjugation of fatty acids, which effectively modifies the drug with lipid moieties.^8–10^ Fatty acids consist of a hydrocarbon chain and a reactive carboxyl group. The mere existence of the carboxyl terminal at the fatty acid part and the hydroxyl group in the oxidized Pt­(IV) analogue allows for covalent conjugation producing a biocleavable bioreduced ester linker at the axial positions of Pt­(IV). A wide range of fatty acids and their derivatives have been explored for such conjugation, including caprylic acid, stearic acid, elaidic acid, and oleic acid (Figure 1). Such modification modulates the lipophilicity of the final platinum­(IV) compound, which enhances its interaction with lipid membranes and improves its compatibility with lipid-based drug delivery systems like liposomes. The value and impact of these delivery platforms in chemotherapy are significant: they are biocompatible, can encapsulate both hydrophilic and hydrophobic compounds, and most importantly, have the potential to prolong drug circulation time while reducing systemic toxicity.^11–15^ By modulating the incorporation of platinum drugs into these carriers, fatty acid conjugation offers a promising path to developing platinum-based therapies that are safer, more targeted, and ultimately more effective. This fundamentally improves how the body handles the drug, leading to a significant impact on cancer treatment and therapeutic outcomes.

**Figure 1:**
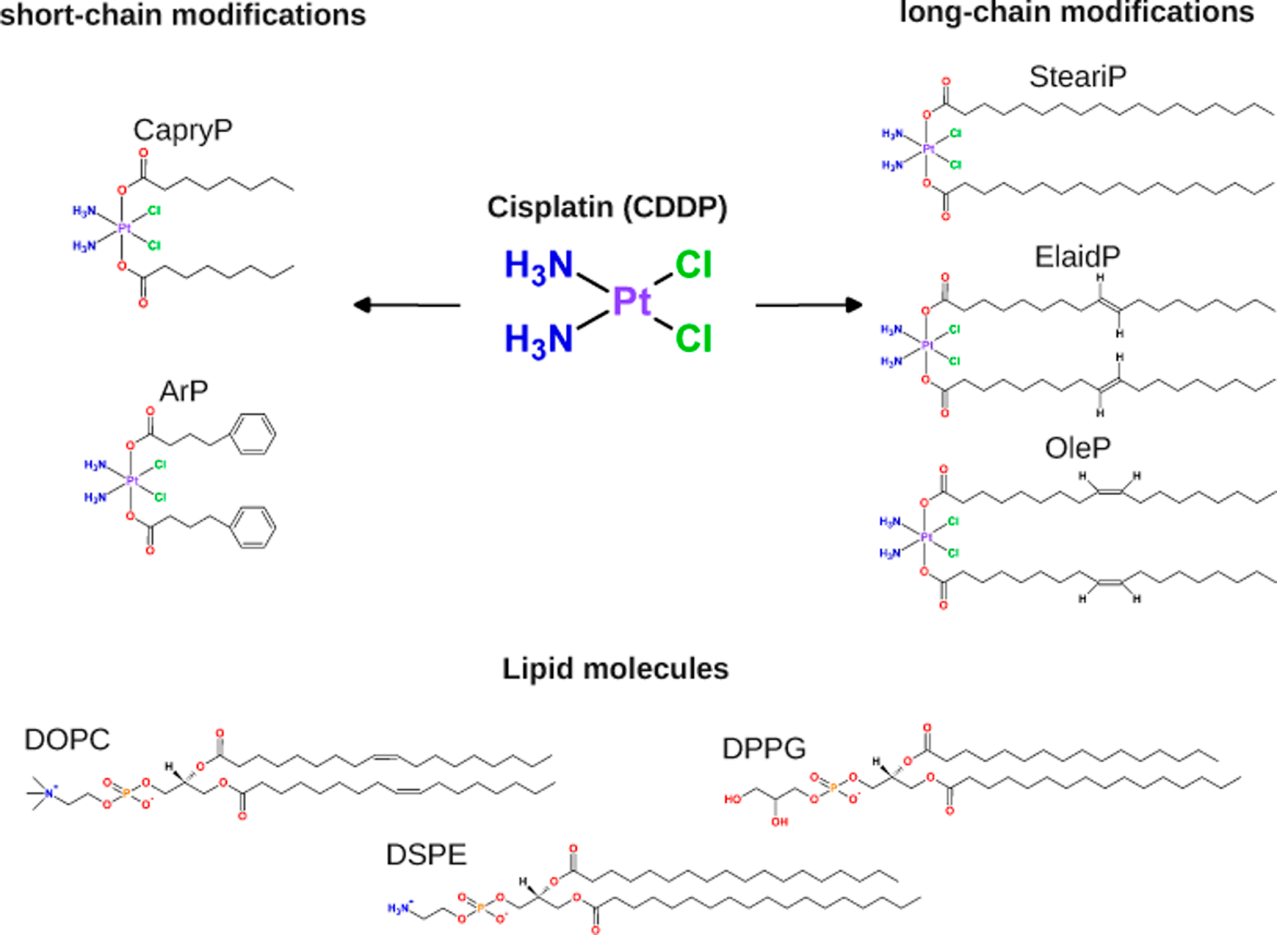
Chemical structures of: (top) Pt-based compounds modified with fatty acid chains of varying length and functionality. CDDP refers to cisplatin, while the other acronyms indicate specific fatty acid or ligand conjugations: CapryP represents caprylic acid (C8) conjugation, ArP denotes an aromatic ring modification, SteariP corresponds to stearic acid (C18:0), ElaidP to *trans*-elaidic acid (C18:1), and OleP to *cis*-oleic acid (C18:1), (bottom) lipid molecules including DOPC, DPPG and DSPE.

Although fatty-acid–conjugated Pt­(IV) prodrugs have been successfully encapsulated in polymeric nanoparticles,^7,16^ it remains unclear how ligand structure, lipophilicity, and lipid composition govern their incorporation and stability in liposomal formulations.^17^ Furthermore, the molecular features underlying drug-lipid interactions in such systems have not been explored systematically. Given these gaps, atomistic molecular dynamics (MD) simulations provide a powerful approach to investigate these interactions at the molecular-level and to guide rational formulation design.^18^

In this study, we employ atomistic molecular dynamics (MD) simulations to investigate the interactions between Pt-based compounds and lipid molecules. The compounds studied include cisplatin, also known as *cis*-diamminedichloroplatinum­(II) (CDDP), and a series of fatty acid conjugates: CapryP (conjugated with caprylic acid, C8), ArP (conjugated with an aromatic ring), SteariP (conjugated with stearic acid, C18:0), ElaidP (conjugated with elaidic acid, C18:1, trans), OleP (conjugated with oleic acid, C18:1, cis)^19^ (see Figure 1). The lipid molecules, phosphatidylcholine (DOPC), phosphatidylglycerol (DPPG), and phosphatidylethanolamine (DSPE), were selected as representative components of liposomal formulations used for Pt-based drug delivery:^20–22^ DOPC provides a fluid PC-rich matrix, DSPE contributes to formulation stability, and DPPG introduces an anionic character relevant to drug–lipid electrostatic interactions. Simulating early aggregation between these lipids and fatty acid Pt­(IV)-conjugate prodrug is rationalized by the expectation that the fatty tails engage in intermolecular interactions. This approach enables the investigation of how the structural features of both ligands and lipids determine their incorporation and stability in nanocarriers. Our findings underscore how the hydrophobic chains of SteariP, ElaidP, and OleP promote drug-lipid interactions, facilitating effective incorporation into lipid assemblies. This study provides a molecular-level perspective on how Pt-based compounds interact with lipid assemblies, offering insights that could guide the rational design of more effective and targeted chemotherapeutic formulations.

## Results

### Dynamics of the Self-Assembly into Micelle Like Structures

During simulations, the lipid molecules and Pt-based compounds spontaneously assembled into micelle-like aggregates, with hydrophilic head groups facing the aqueous environment and hydrophobic tails directed inward. To investigate this behavior, we performed cluster analysis throughout the MD simulations (see Figure 2). A sharp initial drop in the number of clusters indicated rapid aggregation, followed by stabilization into a dominant cluster. Coalescence began as early as 20 ns in some systems, while others formed stable aggregates closer to 200 ns, reflecting variable assembly dynamics. Representative snapshots of the initially formed micelle-like structures are shown in Figures 2 and S1. As shown in Figure 2, Pt-based compounds with fatty acid-conjugation contributed to the transition from fully dispersed molecules to a dominant cluster. In contrast, cisplatin was excluded from the lipid aggregates and formed a separate cluster, resulting in two persistent dominant clusters. This suggests that the assembly is similar to that of pure lipid systems.

**Figure 2:**
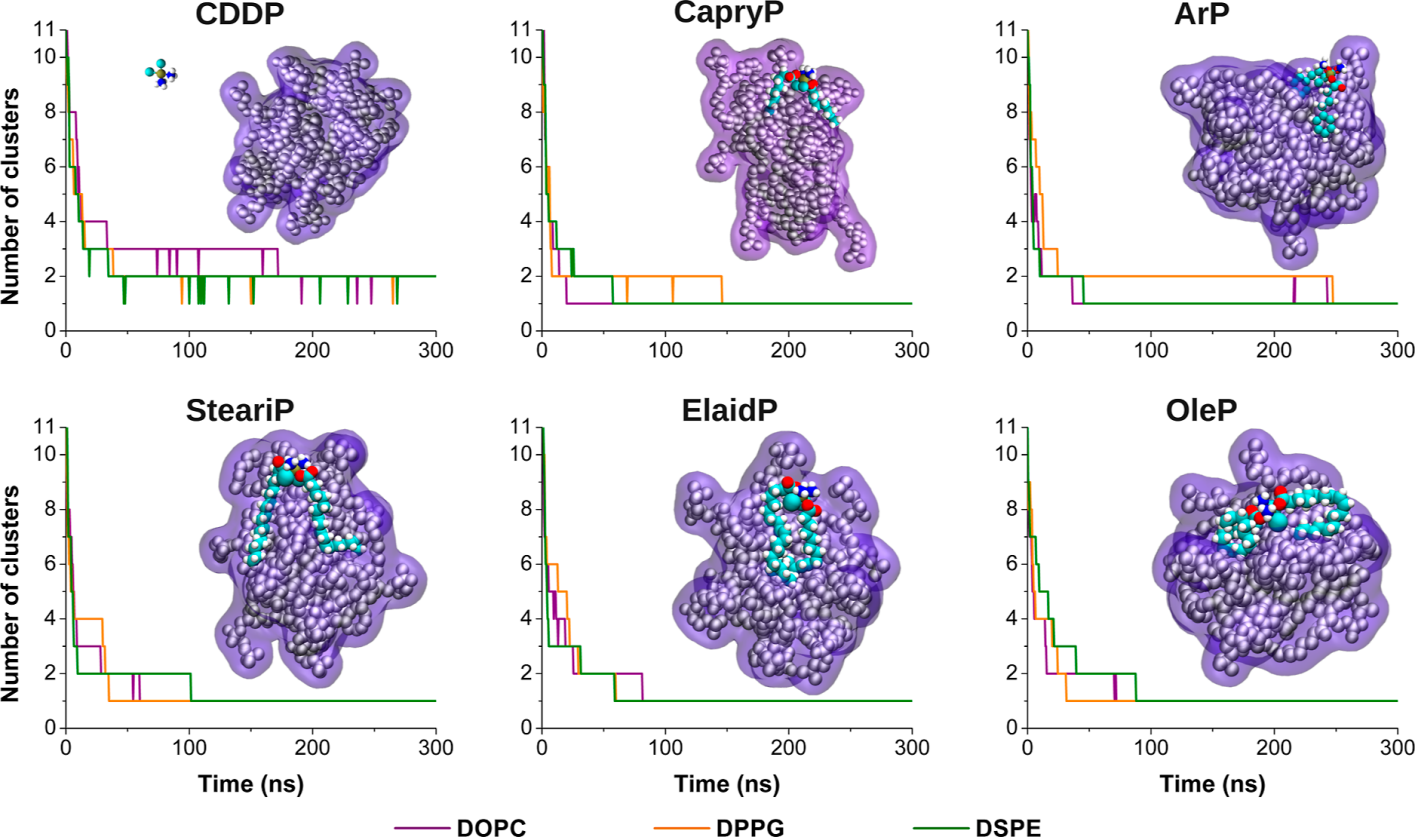
Time evolution of the number of clusters during the self-assembly process of lipid–drug systems. Results are shown for each Pt-based compound (CDDP, CapryP, ArP, SteariP, ElaidP, and OleP) simulated together with different lipids: DOPC (violet), DPPG (orange), and DSPE (green). A representative snapshot of the initially formed micelle-like structure at approximately 200 ns is shown for each system with DOPC lipids. The lipid molecules are shown in van der Waals (VDW) representation and overlaid with a violet surface to highlight the overall micelle-like morphology. Drug molecules are shown in VDW representation and colored by atom type: carbon and chloride in cyan, hydrogen in white, oxygen in red, nitrogen in blue, and platinum in tan. Corresponding structures with DPPG and DSPE are provided in Figure S1.

To probe the role of these compounds in early aggregation, we quantified the number of lipid molecules interacting with each Pt-based compound during the first 200 ns, prior to the formation of compact aggregates as shown in Figure S2. Initial contacts ranged from transient interactions with one or two lipids to more stable associations with three or four lipids lasting tens of nanoseconds. Notably, these early interactions appeared to promote further lipid recruitment, leading to a gradual increase in lipid contacts beyond the initial binding event. Over time, the number of associated lipids stabilized at an average of 5–7 lipids, reflecting progressive embedding of the Pt-based compounds within the aggregate.

Clustering profiles shown in Figure 2 reveal how different Pt-based compounds modulate aggregation in zwitterionic (DOPC and DSPE) and charged (DPPG) lipid environments. CapryP and ArP, which contain medium-length (C8) or aromatic groups, promote rapid formation of micelle-like structures in DOPC. In DPPG, however, the formation of dominant clusters is delayed (∼150–250 ns), possibly due to the reduced lipophilicity of the Pt­(IV) conjugates with shorter fatty chains. Additionally, localized electrostatic interactions at the atomic level, such as those between the carboxyl groups of the Pt-based compounds and lipid headgroups may contribute to this behavior.

Conversely, long-chain conjugates (SteariP, ElaidP, OleP) enabled faster aggregation in DPPG (∼30–60 ns), suggesting that hydrophobic interactions help overcome electrostatic barriers. SteariP, with its saturated tails, promotes rigid, less adaptable aggregation, characterized by the rapid formation of stable clusters with minimal reorganization. This indicates that assembly is driven by energetically favorable configurations rather than a continuous rearrangement. Both ElaidP and OleP, with their unsaturated tails, in contrast, displayed more adaptive self-assembly, with a greater capacity for molecular rearrangement. Despite similar stepwise aggregation behavior, OleP with cis double bonds showed broader steps and long-lived intermediates, indicating gradual and flexible assembly, while, ElaidP with trans double bonds showed sharper, more frequent steps, reflecting faster, more rigid behavior. These differences highlight the role of tail geometry in modulating aggregation pathways, particularly in rigid saturated lipids like DSPE and DPPG, compared to unsaturated DOPC lipid, which better accommodates diverse tail geometries of OleP and ElaidP, resulting in less pronounced differences in the aggregation pathway.

### Key Characteristics of the Micelle-Like Structures

To characterize the micelle-like structures, we first analyzed the mass density profiles relative to the center of mass of the dominant aggregate for DOPC, DSPE, and DPPG lipids (Figure S3), providing a spatial overview of the molecular organization at equilibrium. The profiles show that DOPC exhibited the highest density, followed by DPPG and DSPE, suggesting an ordering of the lipid packing. Notably, the highest peak densities were observed in systems containing cisplatin, CapryP and ArP. For example, the peak value of the mass density profile for DOPC in these systems was 59.6, 59.3, and 58.4 kg/m^3^, respectively. In contrast, systems containing SteariP, ElaidP, and OleP showed lower peak densities of 51.0, 50.9, and 55.9 kg/m^3^, respectively. These observations indicate that smaller platinum-based compounds, those with short carbon chains or aromatic groups, appear to promote the formation of more compact micelle-like structures. Conversely, compounds with longer chains, such as SteariP, ElaidP, and OleP, tend to disrupt the lipid packing, leading to more dispersed aggregates. It is important to note that systems with cisplatin serve as a reference for intact micelle organization, as shown in Figure 2, where cisplatin does not integrate into the micelle-like structure and thus results in a pure lipid micelle.

To monitor the structural evolution over time, we calculated the radius of gyration (*R*_g_) (Figure 3) and the solvent-accessible surface area (SASA) (Figure S4) of the lipid components throughout the simulations. As shown in Figure 3, the *R*_g_ values were initially high due to the dispersed starting configuration. This was followed by significant fluctuations during the first few nanoseconds, reflecting the dynamic rearrangement of lipids and Pt-based compounds as the system progressed toward aggregation. This behavior was particularly pronounced in systems containing cisplatin, especially with DOPC, where cisplatin appeared to be less effective in promoting aggregation compared to fatty acid–conjugated Pt-based compounds, which exhibit a stronger lipid affinity. A similar trend was observed for the DPPG systems with CapryP and ArP, where repulsive interactions were apparent. Despite early fluctuations, all systems showed a gradual decrease in *R*_g_ over time, corresponding to the self-assembly process. By approximately 100 ns, the *R*_g_ values stabilized between 1.24 and 1.32 nm, indicating the formation of a stable micelle-like structure.

**Figure 3:**
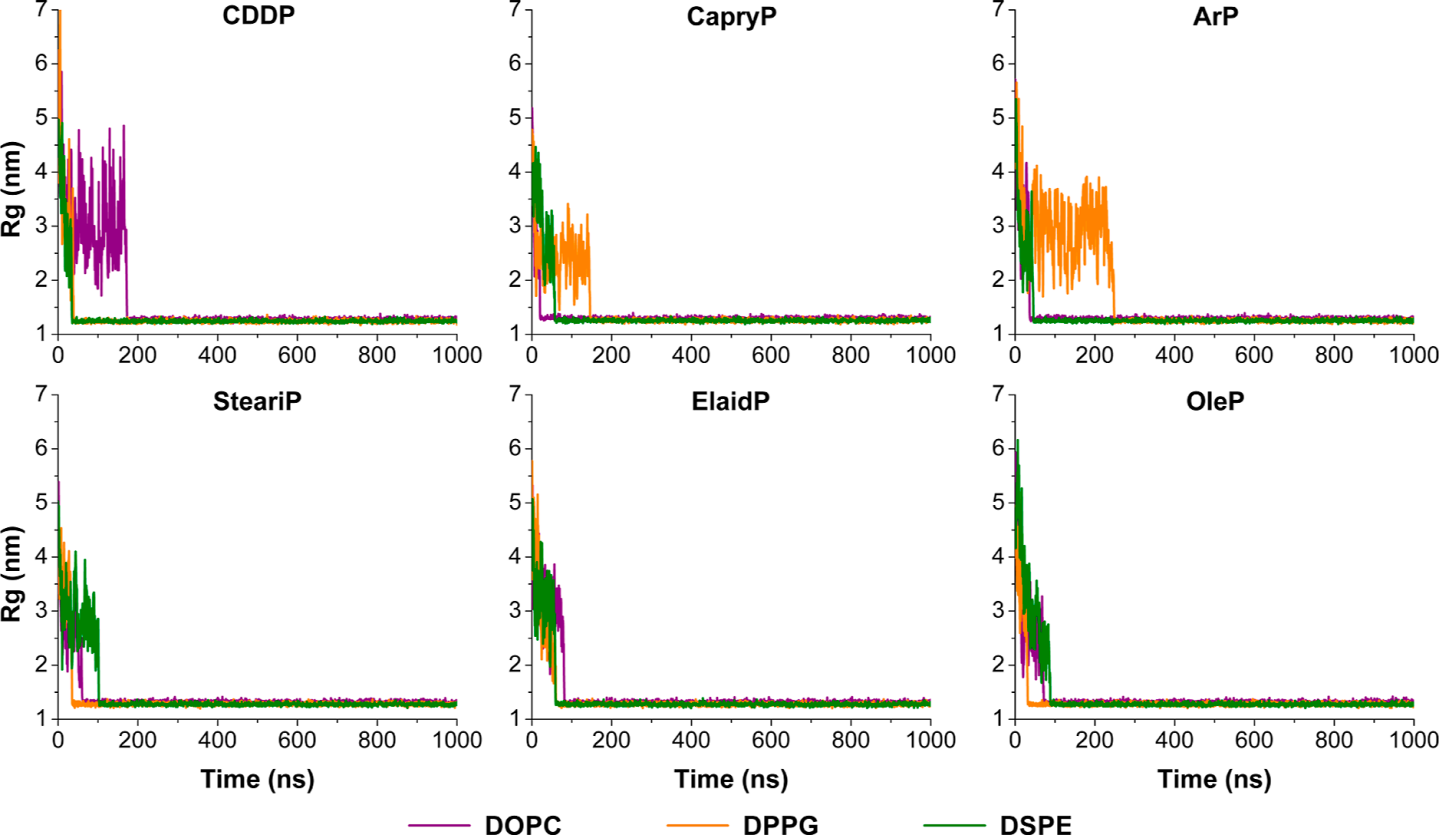
Radius of gyration (*R*_g_) profiles over time reflecting micelle-like formation of DOPC (violet), DPPG (orange), and DSPE (green) in the presence of various Pt-based compounds, as labeled above each panel.

SASA analysis shown in Figure S4 provides complementary insight into micelle stabilization. SASA values decreased during the early stages of simulations, reflecting the shielding of hydrophobic regions from the aqueous environment as the aggregates matured. A stable plateau was reached around the same time as *R*_g_ stabilization occurred, further confirming structural consolidation. In systems containing cisplatin, SASA plateaued at approximately 61, 52, and 53 nm^2^ for DOPC, DPPG, and DSPE lipids, respectively. Slightly higher SASA values were observed in systems containing Pt-based compounds conjugated with saturated and unsaturated fatty acids (SteariP, Elaid, and OleP). This increase reflects partial surface exposure of the hydrophilic moiety of Pt-based compounds, despite stronger hydrophobic interactions mediated by their fatty acid tails. As a result, these systems exhibited more dynamic and less compact micelle-like structures.

### Spatial Proximity and Interaction Strength

Clustering analysis revealed that, unlike cisplatin, the Pt-based compounds were consistently incorporated into the lipid aggregates. To further characterize these interactions, we analyzed the center-of-mass (COM) distances between each compound and the micelle-like structures throughout the simulation (see Figure 4). In the cisplatin systems, the COM distances remained around 5 nm, indicating no stable association with lipid assemblies. Cisplatin moved freely within the simulation box and did not show preferential localization near the micelle-like structure for any of the lipid types, consistent with the clustering results. In contrast, the fatty acid conjugated Pt-based compounds showed significantly shorter and more stable COM distances relative to the lipid assemblies. Long-chain conjugates (SteariP, ElaidP, OleP) maintained COM distances around ∼1.2 nm. Given that the micelle radius ranged from 1.6 to 1.7 nm (see Table S1), these values suggest that the compounds are embedded well within the micelle-like structure. The shorter-chain analogs (CapryP, ArP) exhibited slightly larger COM distances (≈1.4 nm), suggesting a peripheral localization. ArP showed transient excursions to ∼1.6 nm, implying brief dissociation, but consistently returned to the micelle interface, reflecting a weaker yet preferential association.

**Figure 4:**
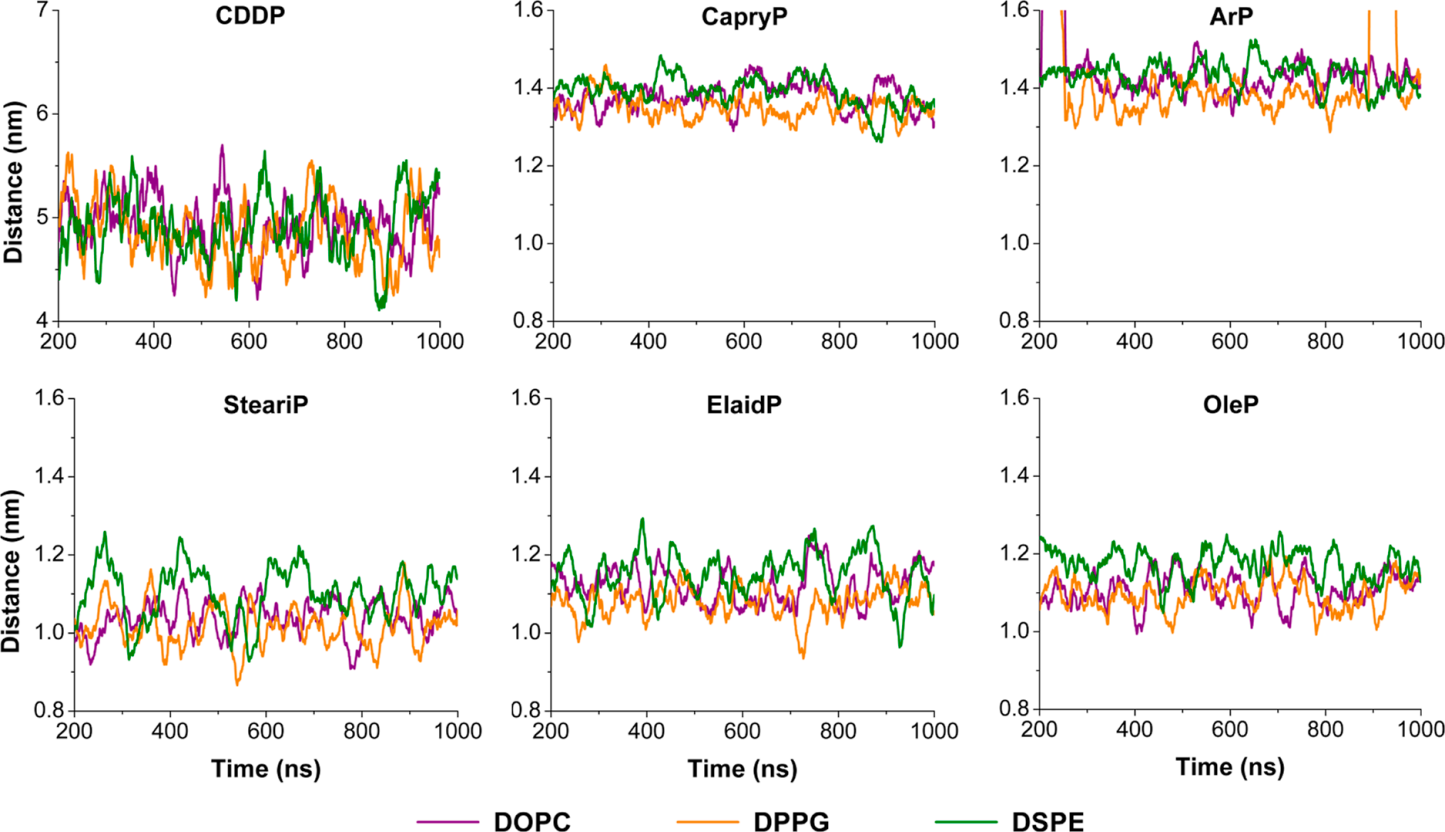
Center of mass (COM) distances between each Pt-based compound (labeled in each panel) and the lipid assemblies composed of DOPC (violet), DPPG (orange), and DSPE (green). The data are only shown for 200 ns onward when micelle formation had occurred in all systems.

To better characterize the embedding geometry for compounds closely interacting with the micelle-like structure, we calculated the angle between each drug’s molecular axis (head to tail) and the vector connecting its COM to that of the micelle-like structure (Figure S5). Long-chain conjugates had low angles (30–45°), indicating deep insertion; shorter analogs showed intermediate angles (50–60°), consistent with interfacial binding. However, the orientation angle alone does not fully capture the internal conformation of the fatty acid tails, which can vary. As illustrated by the snapshots in Figure 5, one or both tails can be extended, folded, or curled within the micelle-like structure and are not necessarily fully extended. To quantify this, we calculated the end-to-end distance between the terminal carbon atom of each fatty acid tail and the platinum atom in the headgroup, and then plotted the difference, Δ*D*, between these two distances (see Figure S5). Short-chain analogs showed near-zero Δ*D*, indicating stably extended tails. Long-chain conjugates exhibited Δ*D* fluctuations (−0.2 to 0.2 nm), reflecting bending or curling of one tail while the other remains extended, suggesting tail flexibility and possible intramolecular interactions or ”breathing motion” with transient exposure to the aqueous environment.

**Figure 5:**
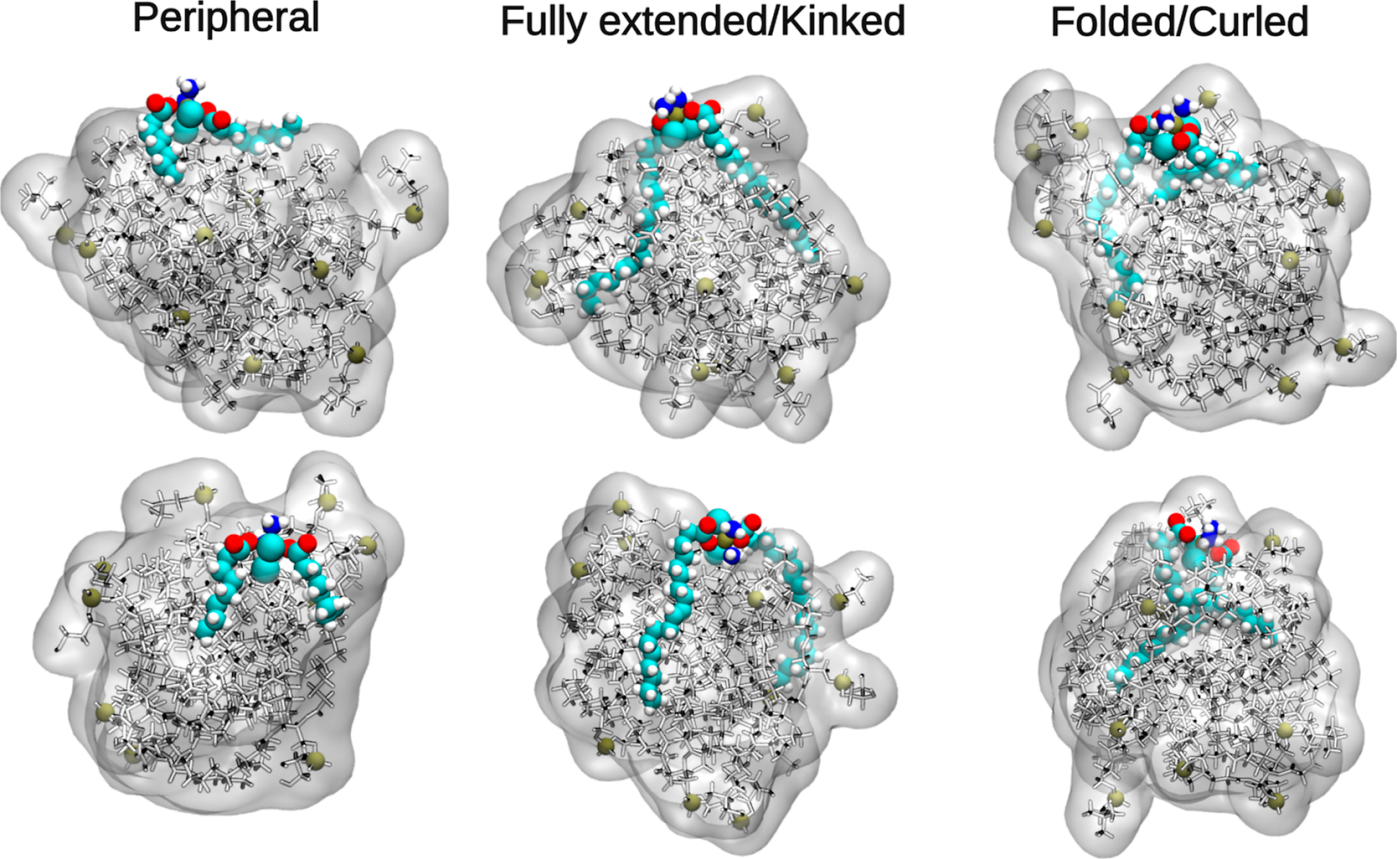
Representative snapshots illustrating the diverse internal conformations of Pt-based compound tails within the micelle-like structure. From left to right, small compounds tend to adopt peripheral orientations with tails extended or slightly tilted, while long-chain fatty acid conjugates exhibit a range of conformations, including extended, kinked, curled, or folded tails. Lipids are shown in stick representation with phosphorus atoms as green spheres marking the headgroups, overlaid by a semitransparent white surface highlighting the micelle-like morphology. The drug molecules are shown in VDW representation and colored by atom name.

To rationalize these findings, we analyzed the total interaction energies between each Pt-based compound and the lipids, as shown in Figure 6. These energies were decomposed into van der Waals and electrostatic contributions (see Figures S6 and S7). As expected, cisplatin showed negligible interaction energy (Figure 6), consistent with its lack of association. In contrast, all fatty acid conjugated compounds showed negative total interaction energies, confirming that their association with lipid assemblies was energetically favorable.

**Figure 6:**
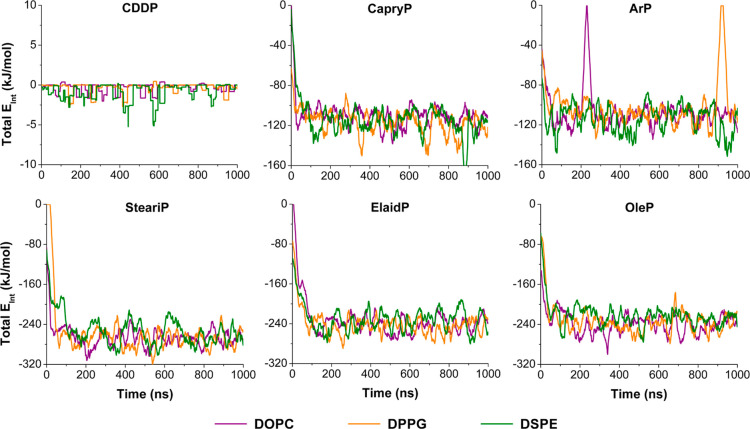
Total interaction energy (van der Waals + electrostatic) calculated between each Pt-based compound (labeled in each panel) and the different lipid types: DOPC (violet), DPPG (orange), and DSPE (green).

Notably, long-chain conjugates (SteariP, ElaidP, OleP) exhibited the strongest interaction energies with the lipids, primarily due to van der Waals contributions (see Figure S6). This underscores the role of hydrophobic insertion in stabilizing lipid association. Electrostatic contributions were significantly weaker, being about 5-fold lower for CapryP and ArP, and about 10-fold lower for SteariP, ElaidP, and OleP. SteariP, with its saturated fatty acid tails, exhibited the strongest van der Waals interactions due to tighter, more ordered packing with the lipid hydrocarbon chains, which maximizes van der Waals contacts. In contrast, the unsaturated tails of ElaidP and OleP introduced conformational kinks that slightly disrupted packing efficiency and reduced hydrophobic contacts.

### Molecular Level Analysis: Distance Map, Contacts and Hydrogen Bonding

To further deepen our understanding of the molecular-level interactions between the Pt-based compounds and lipid assemblies, we analyzed distance maps and contact patterns focusing on specific atomic groupings within the compounds and lipids. The heatmaps in Figures 7 and S8 display average minimum distances between functional groups of the Pt-based compounds (Pt, N, H, Cl, C=O, Pt–O, alkyl chain) and distinct lipid regions (headgroups and tails). Cisplatin, consistent with previous findings, remained spatially distant from lipid assemblies, showing minimal interactions with lipid headgroups at distances of ≥2.5 nm and complete exclusion from micelle-like structures.

**Figure 7:**
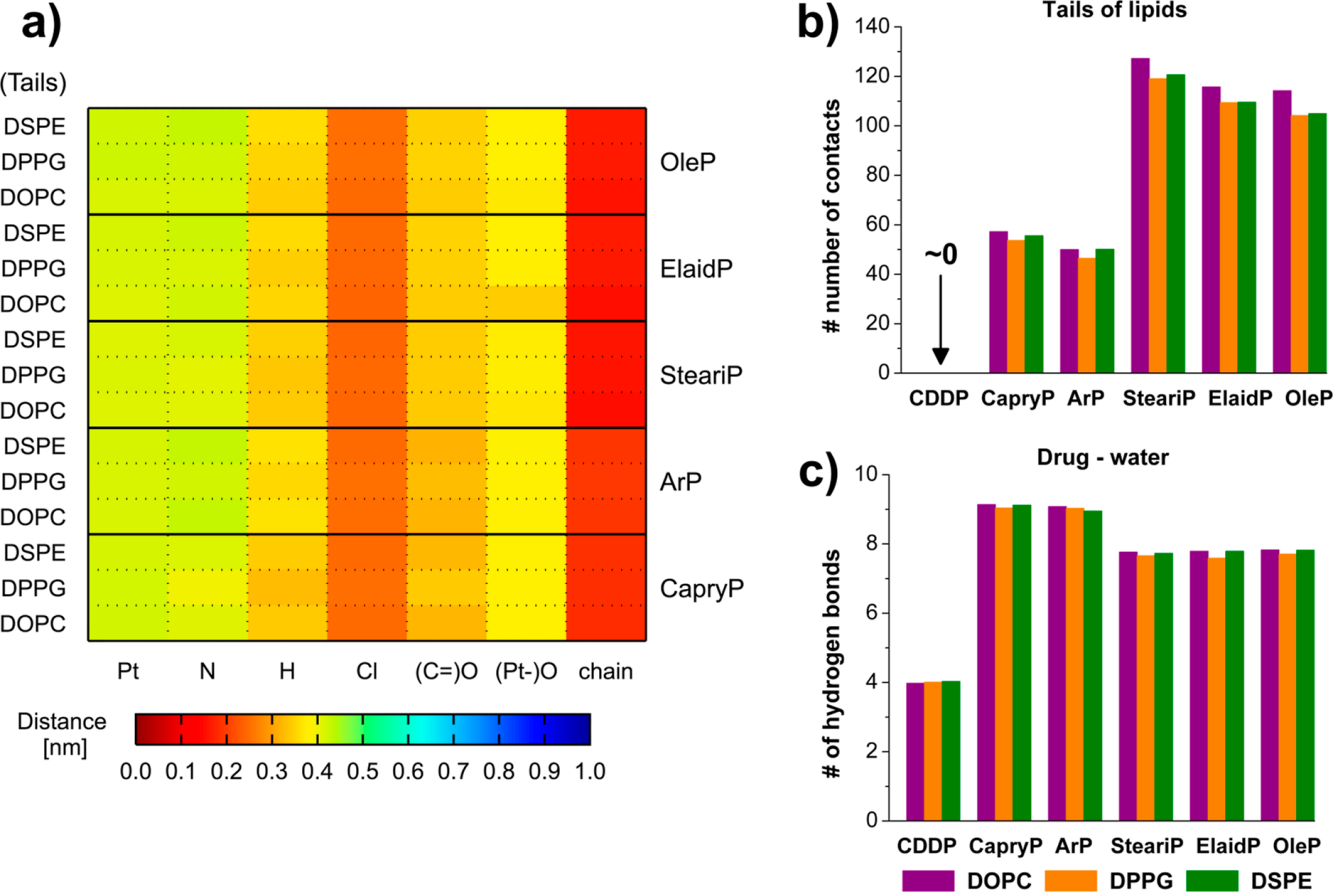
(a) Heatmap of the distance matrix showing contacts between functional groups of the different Pt-based compounds (*x*-axis) and the tails of different lipid types (*y*-axis). The color bar below represents average distances (in nanometers). (b) Average number of heavy atom contacts between each Pt-based compound (*x*-axis) and lipid tails. (c) Average number of hydrogen bonds formed between each Pt-based compounds (*x*-axis) and water molecules. In panels (b,c), lipid types are colored: DOPC (violet), DPPG (orange), and DSPE (green).

In contrast, fatty acid conjugated compounds exhibited significantly closer and more persistent interactions, particularly between their hydrophobic chains and lipid tails. The Chlorine atoms maintained distances within 0.25 nm of the lipid environment, while hydrogens from the ammonia groups and oxygens from the carbonyl/carboxylate groups remained within 0.4 nm (Figure 7a). The relatively rigid coordination geometries around the platinum center appear to constrain the interaction interface, favoring localized binding to specific regions within the lipid core.

Contact analysis further confirmed these observations (see Figure 7b). Cisplatin exhibited negligible lipid contacts (less than one per molecule). In contrast, the fatty-acid conjugated Pt-based compounds formed substantial contacts with the lipid tails. SteariP, ElaidP, and OleP formed an average of more than 100 up to 120 atomic contacts (not counting H atom contacts). This highlights the important role of the alkyl chain length and hydrophobicity in promoting the deep embedding of Pt-based compounds within the micelle-like structures.

Additional insight into the hydration and partitioning behavior was obtained from the analysis of hydrogen bonding with water (see Figure 7c). Cisplatin exhibited an average of four hydrogen bonds with water, consistent with its small size and limited number of polar groups. Pt-based compounds with short alkyl chains or aromatic groups (CapryP and ArP) showed the highest number of hydrogen bonds (∼9), reflecting their greater ability to form H-bonds compared to cisplatin and their greater surface exposure to the aqueous environment and limited embedding within the lipid phase as compared to the long-chain Pt-based compounds. The latter (SteariP, Elaid, and OleP) formed slightly fewer hydrogen bonds (∼8), indicating partial insertion of their hydrophobic tails into the lipid core, with only their polar headgroups accessible for water interactions. These trends, combined with the contact analysis, provide a coherent picture of how hydrophobic insertion and surface exposure balance to determine the localization of Pt-based compounds within the lipid assemblies.

Finally, lipid–water hydrogen bonding remained relatively stable in all systems, following a consistent trend of DPPG > DSPE > DOPC (see Figure 8). This suggests that the incorporation of Pt-based compounds, regardless of structural variation, does not significantly disrupt the hydration shell of the lipid headgroups, thus preserving the organization of the micelle-like structures. Similarly, interlipid hydrogen bonding was largely unaffected: DSPE exhibited the highest degree of lipid–lipid hydrogen bonding, followed by DPPG, while DOPC formed virtually none, as it lacks hydrogen donors and therefore cannot participate in lipid–lipid hydrogen bonds. This hierarchy was maintained regardless of whether the system contained cisplatin, short-chain, or long-chain Pt-based compounds, indicating that intrinsic lipid properties, rather than interaction with Pt-based compounds, dominate interlipid hydrogen bonding behavior. For example, DSPE’s saturated chains and compact headgroups favor tight packing and strong interlipid hydrogen bonding, while DOPC’s unsaturated tails and bulky headgroups reduce such interactions.

**Figure 8:**
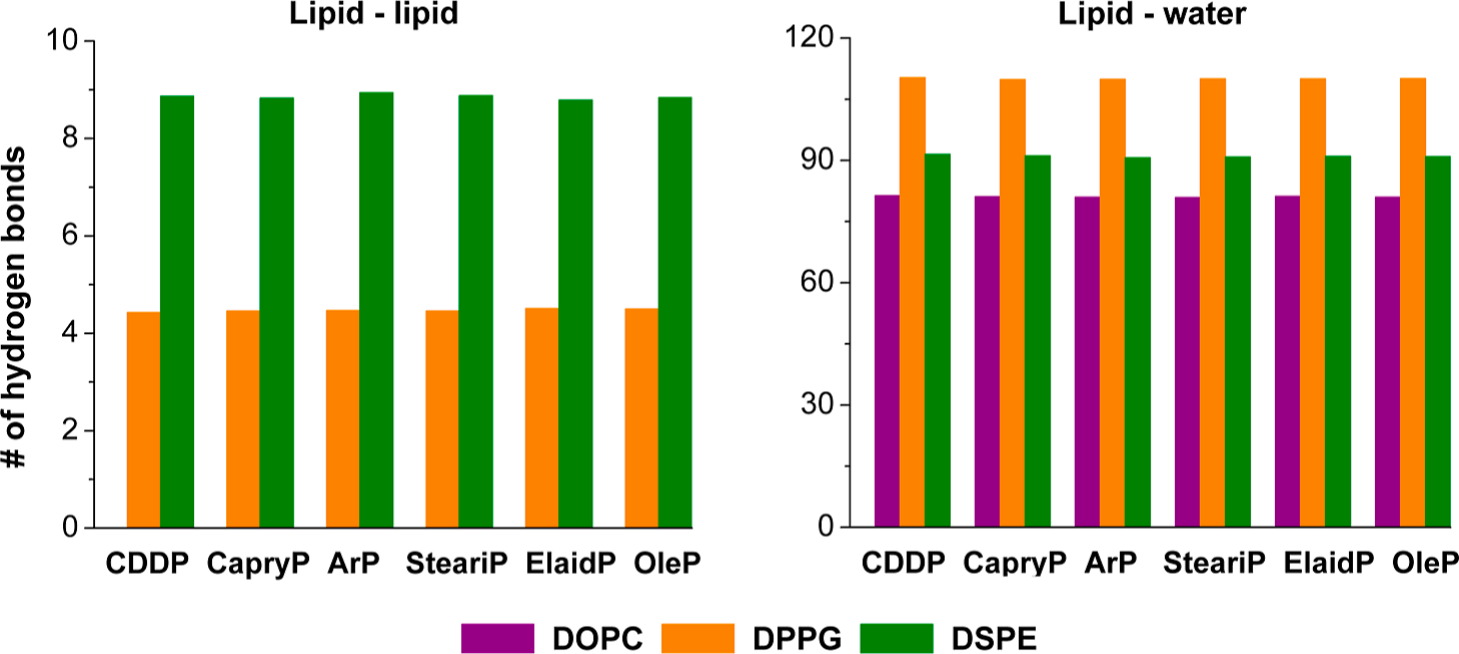
Average number of hydrogen bonds between lipids (left) and between lipid and water (right). This analysis was performed for each simulated system containing different Pt-based compounds, as indicated on the *x*-axis. Lipid types are colored: DOPC (violet), DPPG (orange), and DSPE (green).

## Discussion

In this study, we investigated the interactions between Pt-based compounds and lipid assemblies to better understand their incorporation and behavior within micelle-like structures. To the best of our knowledge, no prior studies have provided quantitative insight into the molecular features governing Pt-based compound incorporation into micelle-like structures. Our results reveal significant differences in lipid affinity between cisplatin and the fatty acid conjugated Pt-based compounds.

Cisplatin, characterized by its small size and limited hydrophobicity, remained excluded from the lipid phase, interacting only minimally with lipid headgroups. This finding is consistent with previous reports highlighting cisplatin’s poor solubility and limited lipophilicity.^23^ By comparison, fatty acid conjugated Pt-based compounds exhibit stronger and more sustained interactions with lipid assemblies, predominantly driven by hydrophobic forces. Compounds with long, saturated chains, such as SteariP, show the strongest van der Waals interactions, promoting stable integration into lipid assemblies. Conversely, compounds with shorter fatty acid chains or aromatic substituents, such as CapryP and ArP, demonstrate weaker lipid interactions and greater aqueous exposure. These findings reveal how chain length and hydrophobicity influence drug-lipid compatibility,^8,24,25^ providing mechanistic insight to guide the rational design of fatty-acid Pt conjugates beyond conventional trial-and-error approaches.

Beyond direct drug-lipid interactions, we also examined the impact of drug incorporation on the structural properties of the lipid assemblies. Importantly, the overall structural integrity of the lipid assemblies remains largely preserved, as evidenced by stable lipid hydration and interlipid hydrogen bonding patterns. These results demonstrate that Pt-based compounds can be stably incorporated without disrupting lipid organization and provide mechanistic insight into their compatibility with the carrier. The preservation of lipid structure is promising for drug delivery applications, where carrier stability is essential.^26–28^

Finally, our results indicate that lipid composition influences drug-lipid interactions.^29,30^ Negatively charged DPPG lipids exhibited stronger associations with Pt-based compounds compared to neutral DOPC, likely due to electrostatic contributions. Furthermore, the inherent flexibility of long fatty acid conjugates supports their stable incorporation into micelle-like structures. To quantitatively capture this conformational behavior, we analyzed both the orientation angle, reflecting the overall alignment, and local conformational metrics, such as the end-to-end distance of the chains. These analyses provide new mechanistic insight into how fatty acid chain dynamics facilitate stable integration into lipid-based systems through bending and reorientation. Together, these findings suggest that selecting specific lipid compositions, in combination with tailored fatty acid ligands, could enable predictive optimization of encapsulation efficiency and stability in liposomal formulations.

## Conclusion

This work provides a molecular-level insight into how Pt­(IV) fatty-acid-like prodrugs interact with and aggregate in phospholipid assemblies composed of DOPC, DPPG, and DSPE. Our simulations identify ligand chain length, unsaturation, flexibility, and lipid composition as key determinants of drug-lipid compatibility and stable incorporation into lipid-based nanocarriers. This mechanistic understanding establishes a foundation for optimizing liposomal loading, stability, and retention, with implications for formulation performance and clinical translation. Importantly, while fatty-acid conjugation has been widely used to enhance the lipophilicity of Pt­(IV) prodrugs, liposomal encapsulation and retention cannot be predicted by lipophilicity alone. Our results demonstrate that effective incorporation depends on a subtle interplay between hydrophobic interactions, ligand flexibility, and lipid headgroup chemistry, highlighting the limitations of simple physicochemical assumptions. By linking molecular aggregation behavior to formulation outcomes, this study provides actionable guidance for the rational, predictive design of Pt-based prodrugs and lipid delivery systems, moving beyond trial-and-error approaches. Although atomistic molecular dynamics simulations cannot fully capture the complexity of biological environments, they offer a powerful framework for dissecting early stage drug–lipid association processes that are difficult to access experimentally. Future studies combining simulations with experimental validation and extending to more complex lipid compositions and longer time scales will further advance the rational development of lipid-based platinum chemotherapeutics.

## Computational Methods

### System Setup and Preparation of Initial Configuration

The interactions of Pt-based compounds with lipid molecules were investigated in a solvated environment using MD simulations. Each Pt-based compound (CDDP, CapryP, ArP, SteariP, ElaidP, and OleP) was simulated with each lipid (DOPC, DPPG and DSPE) at a 10:1 ratio of lipid to Pt-based compounds. Initial configurations were generated using Packmol,^31^ with one Pt-based compound placed at the center of the simulation box and ten lipid molecules distributed randomly around it to avoid bias and allow simulations of the coassembly between the lipids and the respective Pt-based compound. Each resulting system was then prepared for subsequent molecular dynamics simulations by placing it in a 10 nm simulation box and ensuring that the minimum distance between any solute (Pt-based compound or lipid) and the nearest box boundary was 1 nm. The systems were solvated with water, and 150 mM NaCl was added to mimic physiological conditions. Molecular dynamics simulations were performed in GROMACS v 2021.3.^32^ Energy minimization using the steepest descent algorithm^33^ was followed by equilibration: 1 ns NVT at 310 K and 4 ns NPT at 1 bar, using the velocity rescale (v-rescale) thermostat^34^ and the c-rescale barostat,^35^ respectively. Production simulations were run at 1 bar with a 2 fs time step. For each lipid, 1 μs of simulation was performed, resulting in a total of 3 μs per Pt-based compound. Periodic boundary conditions were applied in all directions. Long-range electrostatics were treated with the particle-mesh Ewald (PME)^36^ method, and both electrostatic and van der Waals interactions used a 1.2 nm cutoff.

### Force Field Parameters

Lipid parameters were defined using the lipids force field.^37,38^ Water was modeled using the TIP3P model,^39^ and ion (Na^+^ and Cl^–^) parameters were taken from the Ambertools23 package.^40^ The parameters for the Pt-based compounds were derived using MCPB.py (Metal Center Parameter Builder),^41^ ensuring compatibility with the AMBER force field. MCPB.py generates bonded parameters for transition-metal centers based on quantum mechanical (QM) calculations. The fatty-acid chains were described using a customized GAFF (General AMBER Force Field) parameter set.^42^ The MCPB.py parametrization workflow involved: (i) building 3D structures in GaussView,^43^ (ii) geometry optimization and frequency calculations using Gaussian,^44^ (iii) parameter generation via the Seminario method,^45^ and (iv) Restrained Electrostatic Potential (RESP) charge fitting.^46^ Full QM calculations were performed for cisplatin, CapryP, and ArP. For SteariP, ElaidP, and OleP simplified truncated models were employed to reduce computational cost while preserving the local chemical environment near the platinum center (see Supporting Information Section S1 for details). Dihedral and improper torsions involving the Pt center were set to zero following the MCPB.py protocol.^47^ Lennard–Jones parameters were adopted from the AMBER force field library, and an effective Lennard–Jones radius of 1.22 Å was assigned to the Pt center, consistent with AMBER guidelines for bonded metal ions (effective LJ radius > 1.0 Å) and ensuring stable simulations of all compounds in our set. The resulting AMBER topology (.prmtop) and coordinate (.inpcrd) files were converted into GROMACS-compatible topology and coordinate files (.top and .gro) using the amb2gro_top_gro.py program provided with AmberTools 23 prior to simulation.^40^ Further details of the QM setup and validation of the force field parameters are provided in the Supporting Information.

### Trajectory Analysis

Trajectory analysis was performed using tools from the GROMACS suite, while visualization and snapshot generation were carried out using VMD. Aggregation behavior was assessed with ”gmx clustsize”, and ”gmx distance” was used to calculate the center of mass (COM) distance between Pt-based compounds and lipids. Drug-lipid contacts within a 0.45 nm cutoff were quantified using ”gmx mindist”. Structural metrics such as the radius of gyration (*R*_g_) and solvent-accessible surface area (SASA) were computed using ”gmx gyrate” and ”gmx sasa”, respectively, to assess global compactness and lipid surface exposure. Interaction energies between Pt-based compounds and lipids were calculated using ”gmx energy”. Hydrogen bonding patterns involving drug-water, lipid–water, and lipid–lipid pairs were analyzed using ”gmx hbond”, applying standard geometric criteria (donor (D)–acceptor (A) distance ≤3.5 Å and D–H–A angle ≥120°.

## Supplementary Material



## Notes

Additional figures and tables as described in the text and additional details on quantum mechanical calculations and parametrization of Pt-based compounds (PDF)
