# Supplementary material for: Molecular Insights into the Incorporation of Platinum-Based Drugs into Lipid Aggregates
Source: ACS Omega. 2026 Feb 22;11(9):14869–79. doi: 10.1021/acsomega.5c11203 (PMC12980425; doi:10.1021/acsomega.5c11203)
Supplement: Supplementary file 1 [file ao5c11203_si_001.pdf]

Supporting Information for

"Molecular Insights into the Incorporation of Platinum-based Drugs into Lipid Aggregates"

Kacper Rzepiela,[†,‡] Yousef Najajreh,[¶] Aneta Buczek,[§] Birgit Strodel,[‖,‡] and Hebah Fatafta[*,⊥,‡]

†*University of Opole, Faculty of Chemistry and Pharmacy, Oleska 48, 45-052 Opole, Poland*

‡*Forschungszentrum Jülich, Institute of Biological Information Processing: Structural Biochemistry (IBI-7), Wilhelm-Johnen-Str., 52428 Jülich, Germany*

¶*Al-Quds University, Faculty of Pharmacy, Abu-Dies, P.O.Box 20002, Jerusalem, Palestine*

§*Opole University of Technology, Faculty of Production Engineering and Logistics, Mikołajczyka 5, 45-271 Opole, Poland*

‖*Heinrich Heine University Düsseldorf, Faculty of Mathematics and Natural Sciences, Universitätsstr. 1, 40225 Düsseldorf, Germany*

⊥*Bonn-Rhein-Sieg University of Applied Sciences, Department of Engineering and Communication, Grantham-Allee 20, 53757 Sankt Augustin, Germany*

E-mail: hebah.fatafta@h-brs.de

The supporting information file contains:

Section 1. Quantum Mechanical Calculations and Parameterization of Pt-based compounds.

Table S1. Calculated radii (R_s) of micelle-like structures.

Figure S1. Snapshots of initially formed micelle-like structures for systems with different Pt-based compounds.

Figure S2. Lipid molecules within 0.45 nm of Pt-based compounds during initial aggregation.

Figure S3. Lipid mass density profiles for DOPC, DPPG, and DSPE in systems with different Pt-based compounds.

Figure S4. Time evolution of lipid SASA for DOPC, DPPG, and DSPE in systems with different Pt-based compounds.

Figure S5. Dual y-axis plot showing orientation angle of Pt-based compound relative to micelle COM (left) and difference in fatty acid tail end-to-end distances (right).

Figure S6. Van der Waals interaction energies between Pt-based compounds and DOPC, DPPG, and DSPE.

Figure S7. Electrostatic interaction energies between Pt-based compounds and DOPC, DPPG, and DSPE.

Figure S8. (a) Heatmap of distances, (b) average number of heavy-atom contacts. Both calculated between functional groups of Pt-based compounds and lipid head groups.

Figure S9. 3D Structure of the Pt coordination core in the studied Pt-based compounds.

Section 2. Validation of the force-field parameters.

Figure S10. RMSD as a function of time for the Pt coordination core of the Pt-based compounds simulated in solution.

Table S2. Bond lengths (Å) for the Pt coordination core obtained (MD, MM, QM).

Figure S11. Bond lengths of selected Pt coordination bonds (Pt–N, Pt–Cl, Pt–O) as a function of time.

Figure S12. Bond lengths for the axial Pt-O2 to carbonyl carbon (C2) bond and the carbonyl bond.

Table S3. Bond angles (°) for the Pt coordination core.

Section 1: Quantum Mechanical Calculations and Parameterization of Pt-based Compounds

Quantum mechanical (QM) calculations were carried out using Gaussian 16 software.[1] Geometry optimizations, harmonic frequency analyses, and charge calculations were performed using the long-range corrected CAM-B3LYP functional,[2] which provides a balanced treatment of charge-transfer interactions. The aug-cc-pVTZ basis set[3,4] was used for non-metal atoms, while the Pt atom was treated using the effective core potential (ECP60MDF) with the associated aug-cc-pVTZ-PP basis set.[5] For cisplatin, a more extensive aug-cc-pVQZ/aug-cc-pVQZ-PP level of theory was used to obtain reference data for validation. The GD3BJ dispersion correction[6] was applied in all calculations. Solvent effects were modeled using the conductor-like polarizable continuum model (CPCM),[7] with water as the solvent.

To manage the computational cost of frequency calculations for long-chain conjugates (StheariP, ElaidP, OleP), truncated models were employed. In StheariP, the saturated C18 chain was capped at carbon 9 with a methyl group. For ElaidP and OleP, truncation was applied at carbon 11, beyond the double bond, to preserve the local electronic environment of the unsaturation. Bond and angle force constants in the vicinity of the platinum center were derived directly from the Cartesian Hessian matrix of these truncated models using the Seminario method.[8] For atoms not directly involved in coordination or bonding with the metal center, such as the distal regions of the aliphatic chains, standard bonded parameters from the AMBER force field were assigned. Dihedral and improper torsional parameters involving the platinum center were set to zero in accordance with MCPB.py guidelines,[9] due to the low energy barriers and the limitations of harmonic approximations in representing torsional flexibility around transition metal centers. RESP charges[10] were calculated from the electrostatic potential of optimized geometries. Lennard-Jones parameters were adopted from the Amber force field and uniformly applied to all Pt-based compounds. The platinum was assigned an effective Lennard–Jones radius of 1.22 Å,

consistent with AMBER guidelines for bonded metal ions. This is a typical value for both Pt(II) and Pt(IV) given their minimal variation in ionic size.

Supplementary Table

Table S1: The table shows the micelle-like structure radius (R_s) calculated for each system, as indicated in the table header. R_s was calculated using $\sqrt{\frac{5}{3}}$ Rg, where Rg is the average radius of gyration after clustering.

	Micellar radius (nm)		
Drug name	DOPC	DPPG	DSPE
CDDP	1.65	1.60	1.61
CapryP	1.67	1.62	1.62
ArP	1.67	1.62	1.62
SteariP	1.70	1.65	1.65
ElaidP	1.70	1.65	1.65
OleP	1.70	1.65	1.65

Supplementary Figures

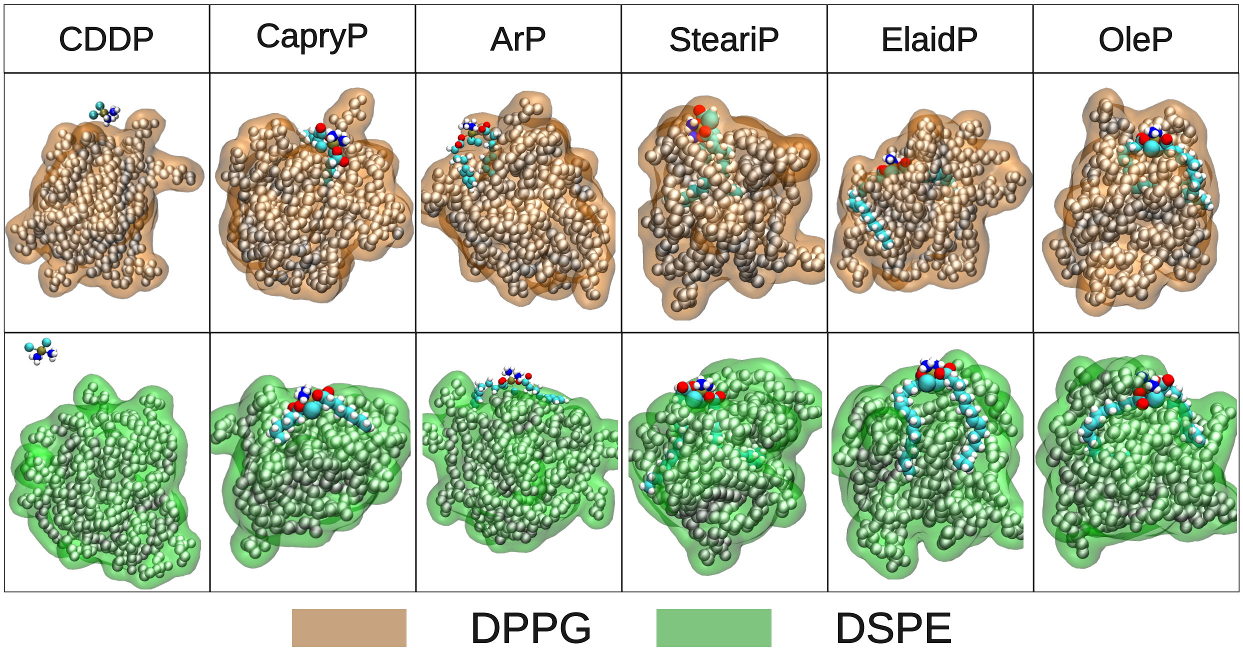

Figure S1: Representative snapshots of the initially formed micelle-like structures at approximately 200 ns. The top row shows snapshots from systems containing DPPG lipids (orange) and the bottom row shows systems with DSPE lipids (green). The corresponding Pt-based compounds (CDDP, CapryP, ArP, SteariP, ElaidP, and OleP) are labeled from left to right, as indicated in the table header. The lipid molecules are shown in van der Waals (VDW) representation and overlaid with an orange or green surface to highlight the overall micelle-like morphology. Drug molecules are shown in VDW representation and colored by atom name.

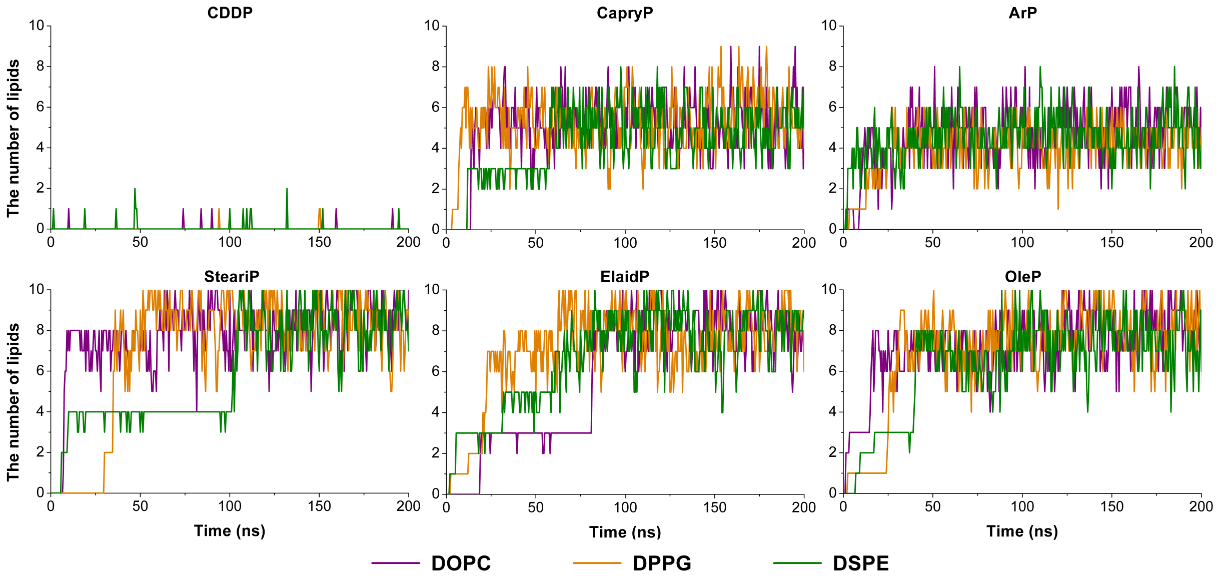

Figure S2: The number of lipid molecules within 0.45 nm of the Pt-based compounds during the initial 200 ns of aggregation, prior to the formation of compact micelle-like structures.

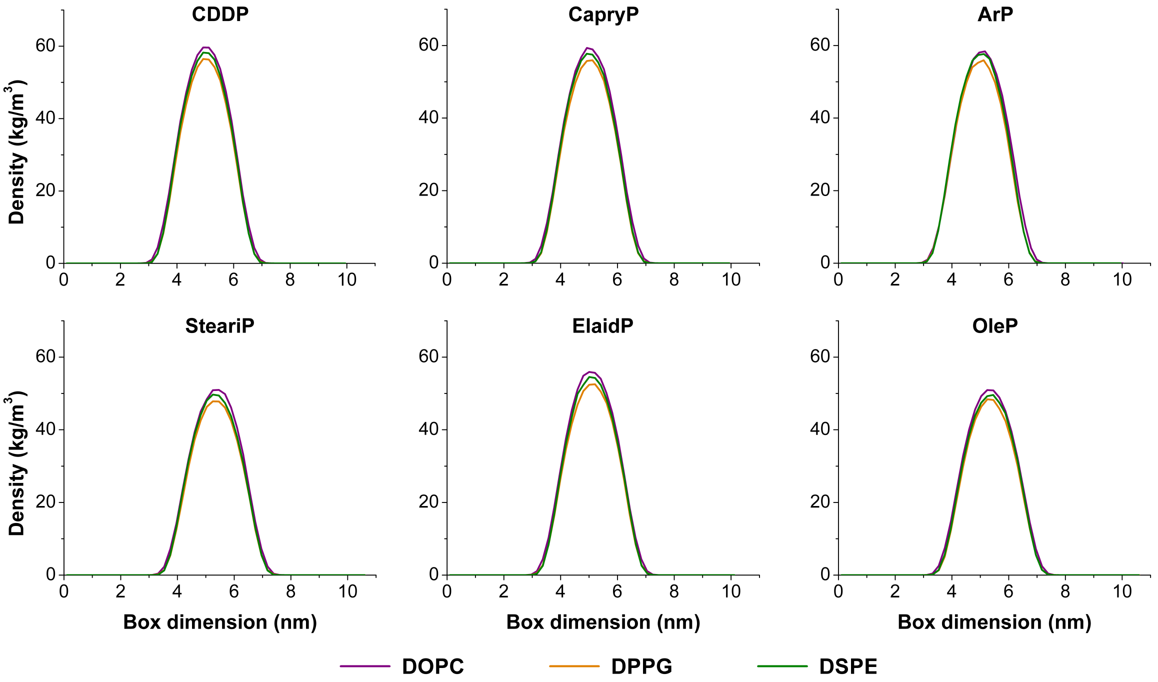

Figure S3: Mass density profiles of DOPC (violet), DPPG (orange), and DSPE (green) for systems containing different Pt-based compounds, as indicated above each panel.

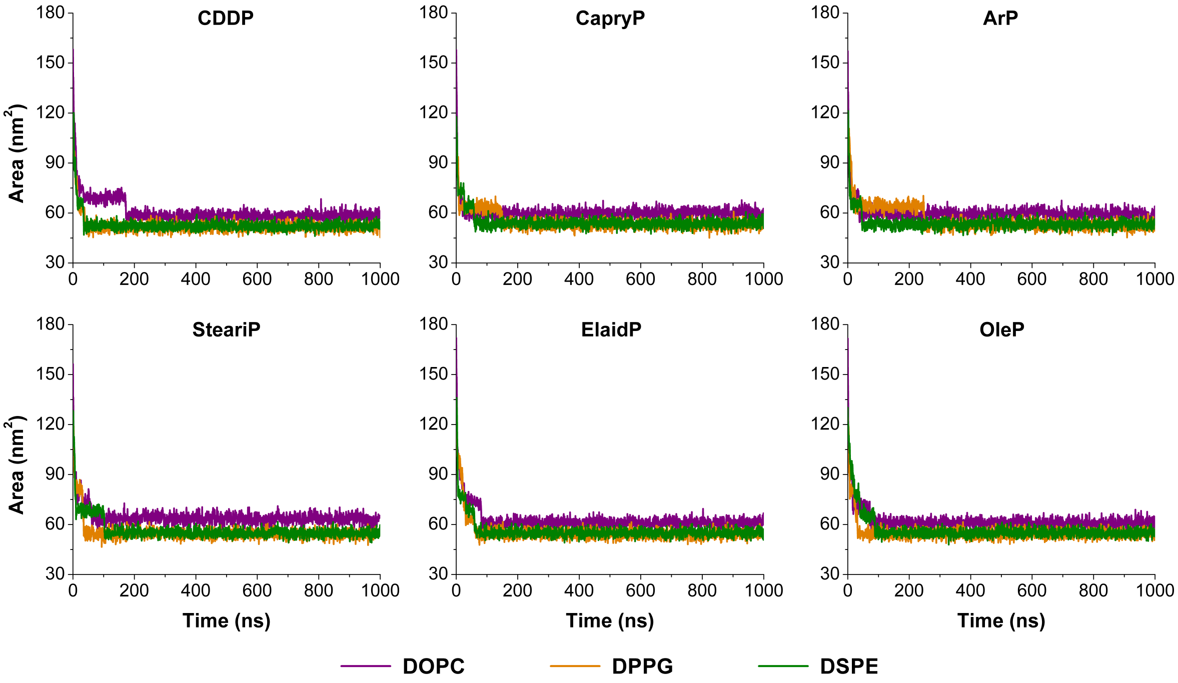

Figure S4: Solvent-accessible surface area (SASA) profiles of lipids over time for DOPC (violet), DPPG (orange), and DSPE (green) in systems containing different Pt-based compounds, as indicated above each panel.

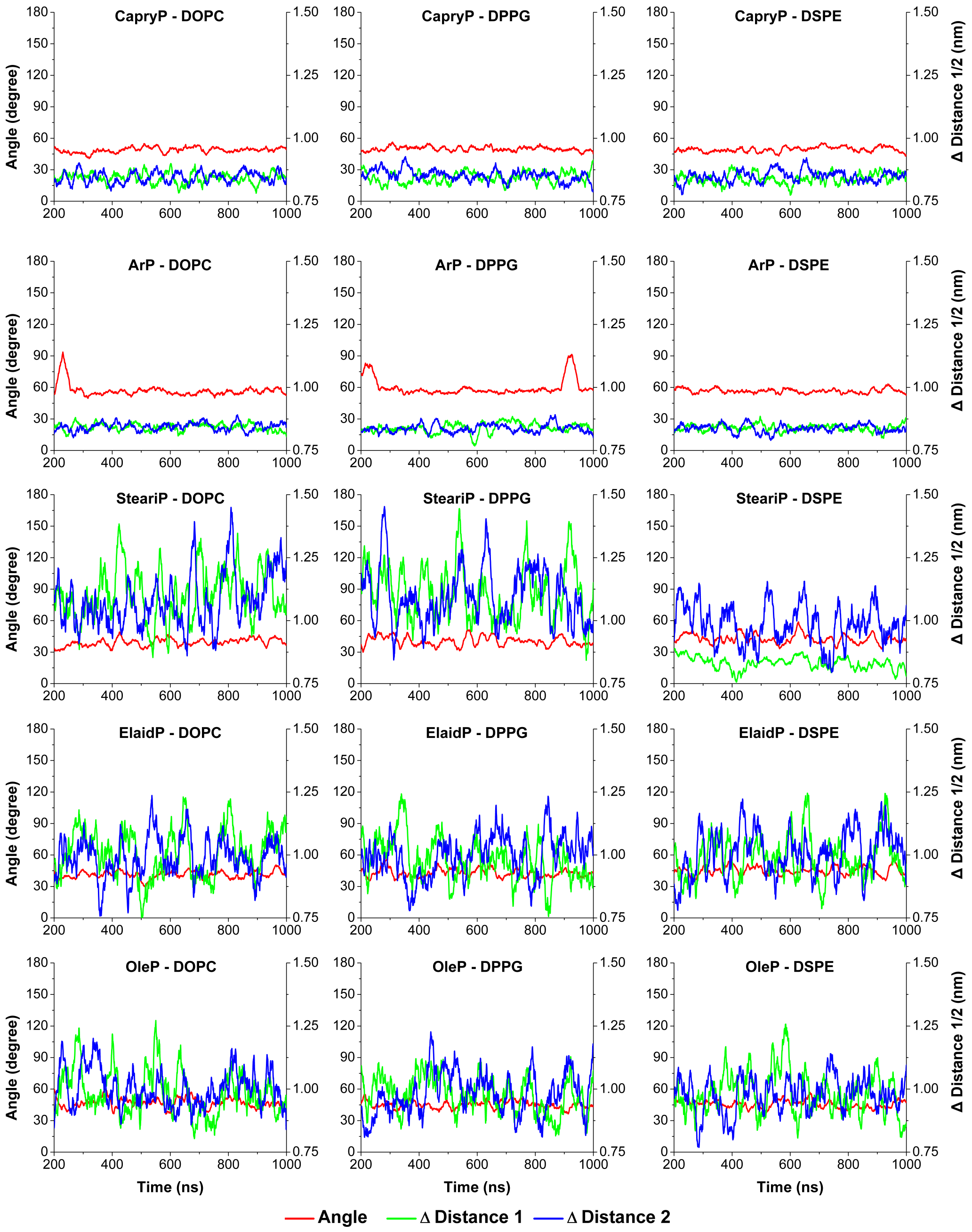

Figure S5: Dual y-axis plot showing, on the left axis, the orientation angle (in degrees) between the vector connecting the center of mass (COM) of the micelle-like structure to that of the Pt-based compound and the molecular axis of the Pt-based compound. The molecular axis is defined from the Pt atom in the head group to the terminal atom of its conjugated fatty acid chain. This angle is calculated for each fatty acid chain, averaged, and shown in red. The right axis displays the difference in end-to-end distances (tail1 – tail2) between the two fatty acid tails of the Pt-based compound, shown in green and blue.

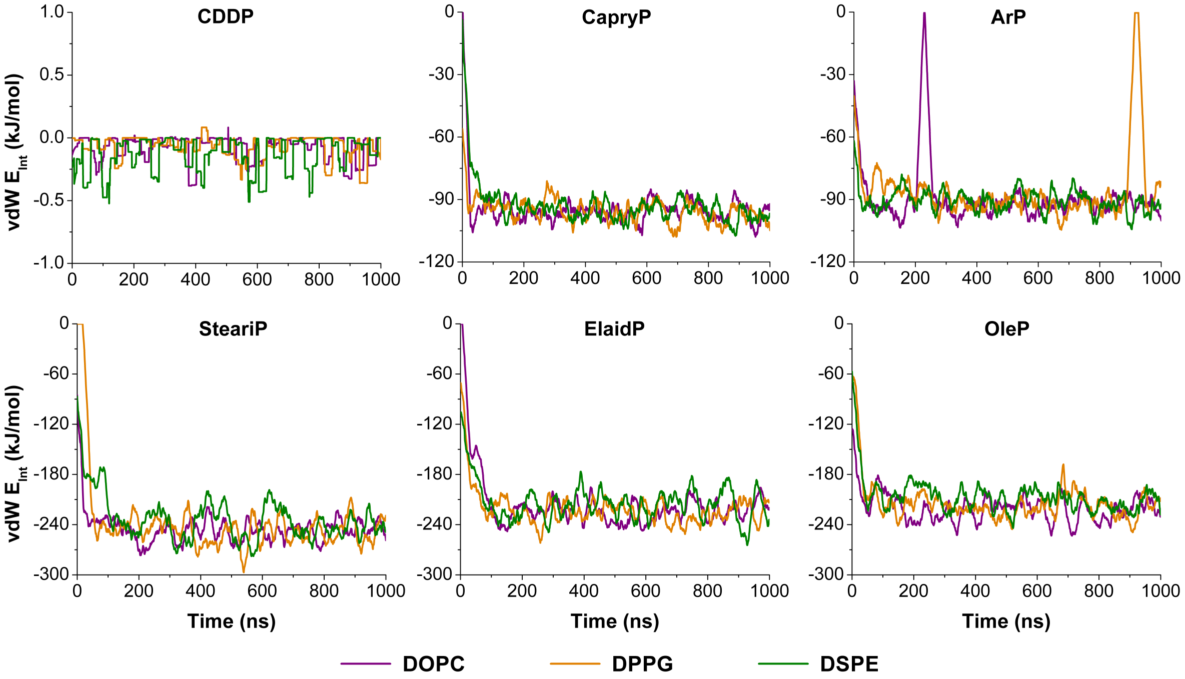

Figure S6: Van der Waals interaction energies calculated between each Pt-based compound (labeled in each panel) and the different lipid types: DOPC (violet), DPPG (orange), and DSPE (green), respectively.

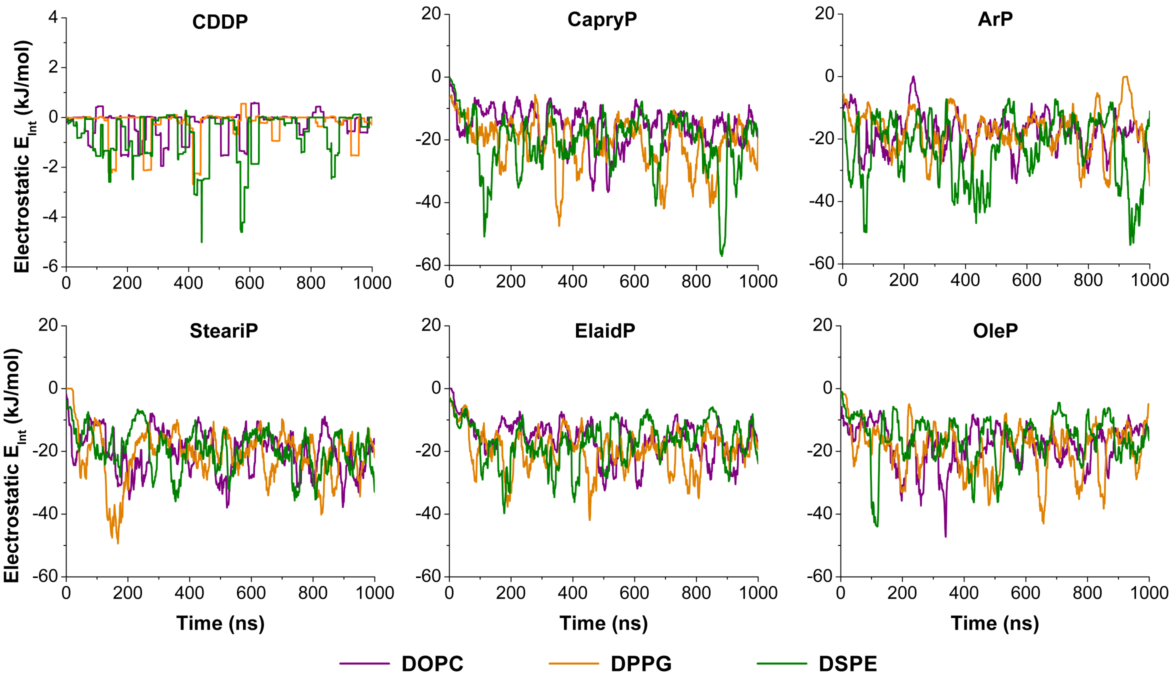

Figure S7: Electrostatic interaction energies calculated between each Pt-based compound (labeled in each panel) and the different lipid types: DOPC (violet), DPPG (orange), and DSPE (green), respectively.

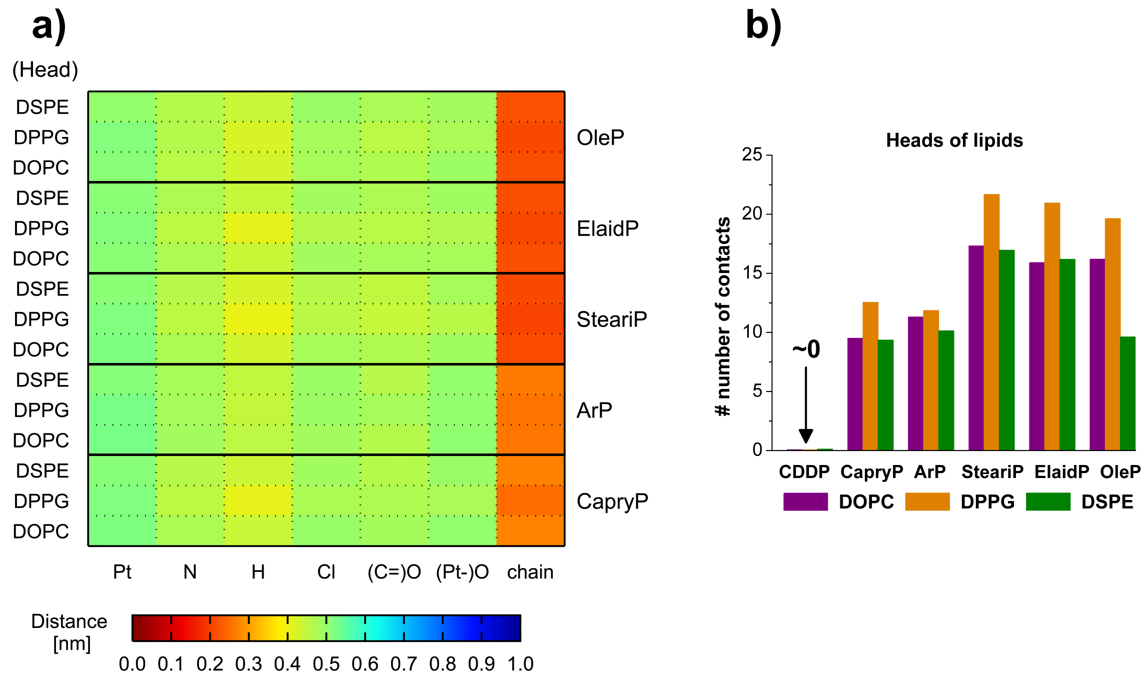

Figure S8: (a) Heatmap of distance matrix showing contacts between functional groups of the different Pt-based compounds (x-axis) and the head groups of different lipid types. The color bar below represents. average distances (in nanometers). (b) Average number of heavy atom contacts between each Pt-based compound (x-axis) and lipid head groups. Lipid types are colored: DOPC (violet), DPPG (orange), and DSPE (green).

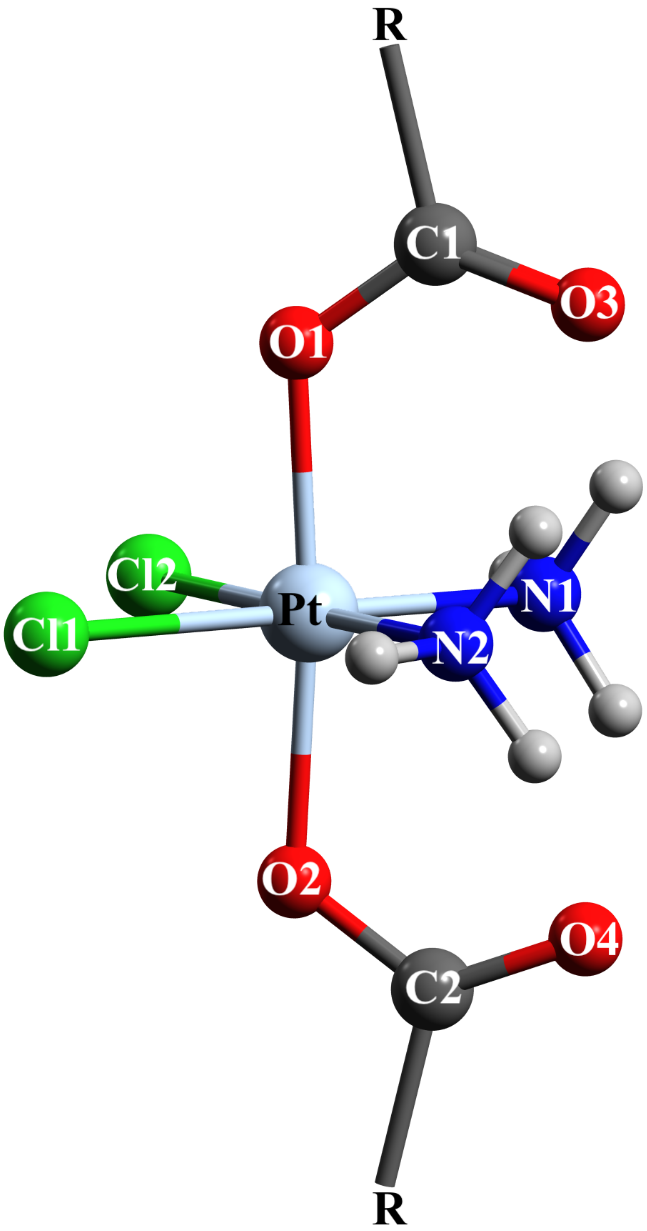

Figure S9: 3D Structure of the Pt coordination core, comprising the platinum atom (Pt) and all directly coordinated atoms in the studied Pt-based compounds. This includes the two ammine ligands (N1, N2), the chloride ligands (Cl1, Cl2), axial oxygen atoms (O1, O2), and the carbonyl carbon (C1, C2) and oxygen ((O3, O4)) of the bound ligand. R represents the fatty acid chains.

Section 2: Validation of the force-field parameters

To validate the Pt-based force-field parameters developed in this work, a two-step validation strategy was employed: (i) molecular mechanics (MM) energy minimization in vacuum, and (ii) molecular dynamics (MD) simulations in solution, with direct comparison to quantum mechanical (QM) reference data. For this purpose, the analysis was restricted to the Pt coordination core generated by MCPB.py, comprising the platinum atom and all directly coordinated atoms, including the two ammine ligands (NH_3), the chloride ligand, axial oxygen atoms, and the carbonyl carbon and oxygen of the bound ligand. The fatty-acid chains were described using

GAFF parameters, which are well established and do not directly affect the metal-ligand bonding. A schematic representation of the defined Pt core is shown in Fig. S9. Details of the calculations and their interpretation are provided below.

(i) Energy minimization: The developed parameter set was first applied in MM energy minimization of the six Pt-based compounds using their QM-optimized geometries as starting structures. Calculations were performed in GROMACS v2018.8 in vacuum. Energy minimization was carried out using the steepest-descent algorithm with an energy tolerance of 100 kJ mol^{-1}, followed by conjugate-gradient minimization with a tightened energy tolerance of 50 kJ mol^{-1} to ensure proper convergence of the molecular geometries.

The resulting MM-minimized structures were compared to the corresponding geometry-optimized conformations from the QM calculations by determining their root mean square deviation (RMSD). The obtained RMSD values were small ($\sim$0.1 Å), indicating close agreement between MM and QM structures. To further quantify the accuracy of the parameters, bond lengths and bond angles within the Pt core were extracted from the MM-minimized structures and compared to the QM reference values (Tables S2 and S3). This validation strategy has been used previously to assess the fidelity of metal-center force-field parameters.[11,12]

As shown in Table S2, the absolute deviations in bond lengths $\Delta = |\mathrm{MM} - \mathrm{QM}|$ do not exceed 0.11 Å, with values ranging from 0.05 to 0.11 Å. Similarly, the deviations in the bond angles range from 0.74° to 3.7° (Table S3). Overall, these results demonstrate that the developed parameters reproduce the equilibrium geometry of the Pt coordination core with good accuracy.

(ii) Molecular dynamics simulations in solution: To further validate the stability of the proposed force-field parameters, MD simulations were performed for each Pt-based compound in explicit TIP3P water for 100 ns.

The time evolution of the RMSD of the Pt coordination core was monitored throughout the simulations (Fig. S10). RMSD remained stable within approximately 0.5-1.0 Å for all complexes, indicating modest structural fluctuations. When the carbonyl carbon atoms are excluded, the Pt center and its directly coordinating atoms (N1, N2, Cl1, Cl2, O1, O2) exhibit extreme stability, with RMSD values of 0.05-0.10Å, confirming the robustness of the Pt coordination environment under the applied force-field parameters. The minor fluctuations observed primarily reflect thermal motion and the inherent flexibility of the axial oxygen–carbonyl moiety, without causing any distortion of the Pt coordination core.

In addition, the temporal behavior of bond lengths and bond angles around the Pt center was analyzed. The average bond lengths exhibited standard deviations between 0.02 and 0.05 Å (Table S2), reflecting only minor thermal fluctuations around the QM reference values. The plot showing the time evolution of the bond lengths over the course of the simulation reveals small fluctuations, but no systematic drift or distortion of the coordination geometry (see Fig. S11 and Fig. S12). Similarly, the average bond angles showed standard deviations in the range of 1.68° to 2.48° (Table S3), with the QM values consistently falling within the sampled distributions. These results further confirm that the Pt coordination environment remains stable during long-timescale MD simulations.

Together, the MM minimization and explicit-solvent MD simulations demonstrate that the parameter set developed using the MCPB.py tool reliably reproduces both the equilibrium geometry and dynamical stability of the Pt coordination core across all investigated compounds.

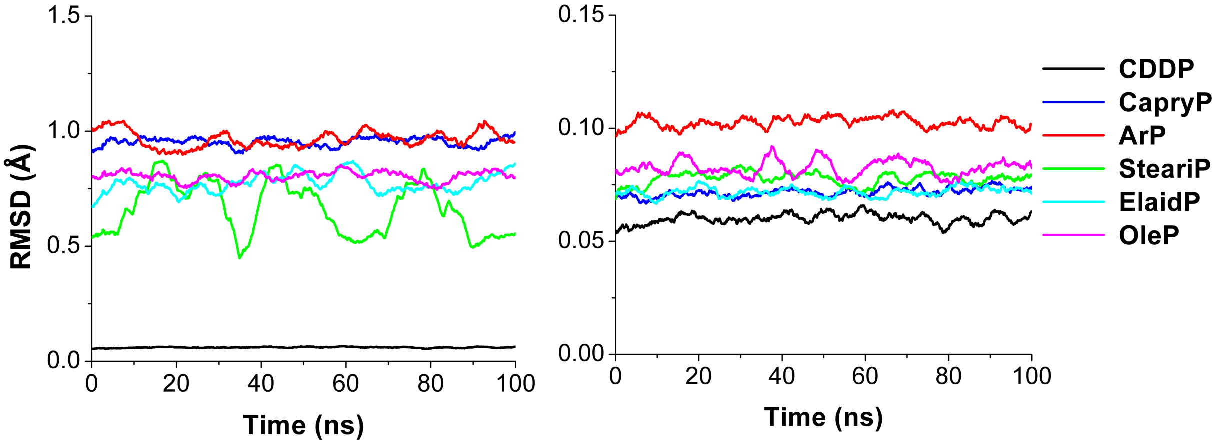

Figure S10: RMSD of the Pt coordination core over time for all Pt-based compounds simulated in solution at 310 K. The initial structure (first frame of the simulation) was used as the reference for RMSD calculations. Left: RMSD of the full Pt coordination core, including the axial carbonyl carbons. Right: RMSD of the Pt coordination core excluding the carbonyl carbons, highlighting the stability of the directly coordinating atoms (N1, N2, Cl1, Cl2, O1, O2).

Table S2: Bond lengths (Å) for the Pt coordination core obtained from (1) MD simulations, reported as average values with standard deviations in parentheses; and (2) MM energy minimization, reported as single-point values with absolute deviations from the QM reference in parentheses $\Delta = |\text{MM} - \text{QM}|$; and (3) QM optimized structures.

System	Method	Pt–N1	Pt–N2	Pt–Cl1	Pt–Cl2	Pt–O1	Pt–O2	O1–C1	O2–C2	C1–O3	C2–O4
CDDP	MD	1.97 (0.05)	1.97 (0.05)	2.27 (0.06)	2.27 (0.05)						
	MM	1.95 (0.10)	1.95 (0.10)	2.25 (0.08)	2.25 (0.08)						
	QM	2.05	2.05	2.33	2.33						
CapryP	MD	1.94 (0.05)	1.94 (0.05)	2.28 (0.05)	2.28 (0.05)	1.93 (0.04)	1.94 (0.04)	1.31 (0.03)	1.31 (0.03)	1.32 (0.03)	1.32 (0.03)
	MM	1.94 (0.11)	1.94 (0.11)	2.27 (0.05)	2.27 (0.06)	1.94 (0.06)	1.94 (0.06)	1.30 (0.00)	1.30 (0.00)	1.32 (0.09)	1.32 (0.09)
	QM	2.05	2.05	2.32	2.33	2.01	2.01	1.30	1.30	1.23	1.23
ArP	MD	1.94 (0.05)	1.94 (0.05)	2.28 (0.05)	2.28 (0.05)	1.94 (0.04)	1.94 (0.04)	1.31 (0.03)	1.31 (0.03)	1.32 (0.03)	1.32 (0.03)
	MM	1.94 (0.11)	1.94 (0.11)	2.27 (0.05)	2.26 (0.06)	1.94 (0.06)	1.95 (0.05)	1.30 (0.00)	1.30 (0.00)	1.31 (0.08)	1.31 (0.09)
	QM	2.05	2.05	2.32	2.33	2.01	2.01	1.30	1.30	1.23	1.23
SteariP	MD	1.94 (0.05)	1.94 (0.05)	2.29 (0.05)	2.28 (0.05)	1.94 (0.04)	1.94 (0.04)	1.21 (0.02)	1.21 (0.02)	1.22 (0.02)	1.22 (0.02)
	MM	1.94 (0.11)	1.94 (0.10)	2.27 (0.06)	2.26 (0.06)	1.94 (0.06)	1.94 (0.06)	1.20 (0.10)	1.20 (0.10)	1.23 (0.00)	1.22 (0.01)
	QM	2.05	2.05	2.33	2.33	2.01	2.01	1.30	1.30	1.23	1.23
ElaidP	MD	1.94 (0.05)	1.95 (0.05)	2.28 (0.05)	2.28 (0.05)	1.94 (0.04)	1.94 (0.04)	1.21 (0.02)	1.21 (0.02)	1.22 (0.03)	1.22 (0.02)
	MM	1.94 (0.11)	1.94 (0.11)	2.27 (0.05)	2.26 (0.06)	1.95 (0.05)	1.94 (0.06)	1.21 (0.10)	1.21 (0.10)	1.22 (0.01)	1.22 (0.01)
	QM	2.05	2.05	2.33	2.32	2.01	2.01	1.30	1.30	1.23	1.23
OleP	MD	1.94 (0.05)	1.94 (0.05)	2.28 (0.05)	2.28 (0.05)	1.94 (0.05)	1.94 (0.04)	1.21 (0.02)	1.21 (0.02)	1.22 (0.02)	1.22 (0.02)
	MM	1.95 (0.10)	1.94 (0.10)	2.27 (0.06)	2.27 (0.05)	1.95 (0.05)	1.95 (0.05)	1.20 (0.10)	1.20 (0.10)	1.22 (0.01)	1.22 (0.01)
	QM	2.05	2.05	2.33	2.32	2.01	2.01	1.30	1.30	1.23	1.23

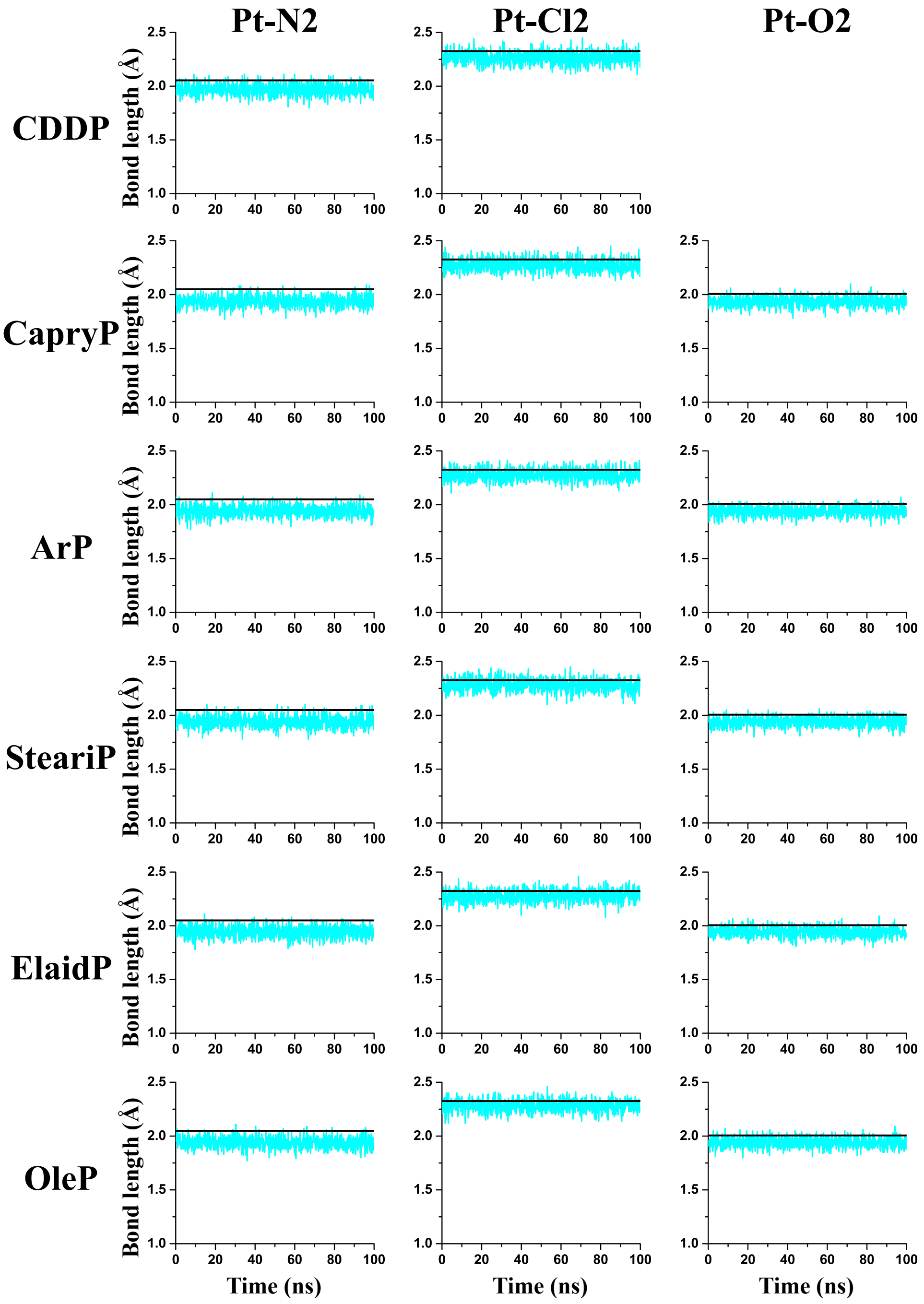

Figure S11: Bond lengths of selected Pt coordination bonds (Pt–N, Pt–Cl, Pt–O) as a function of time over 100 ns of simulation. Shown are Pt–N2, Pt–O2, and Pt–Cl2 as representative examples

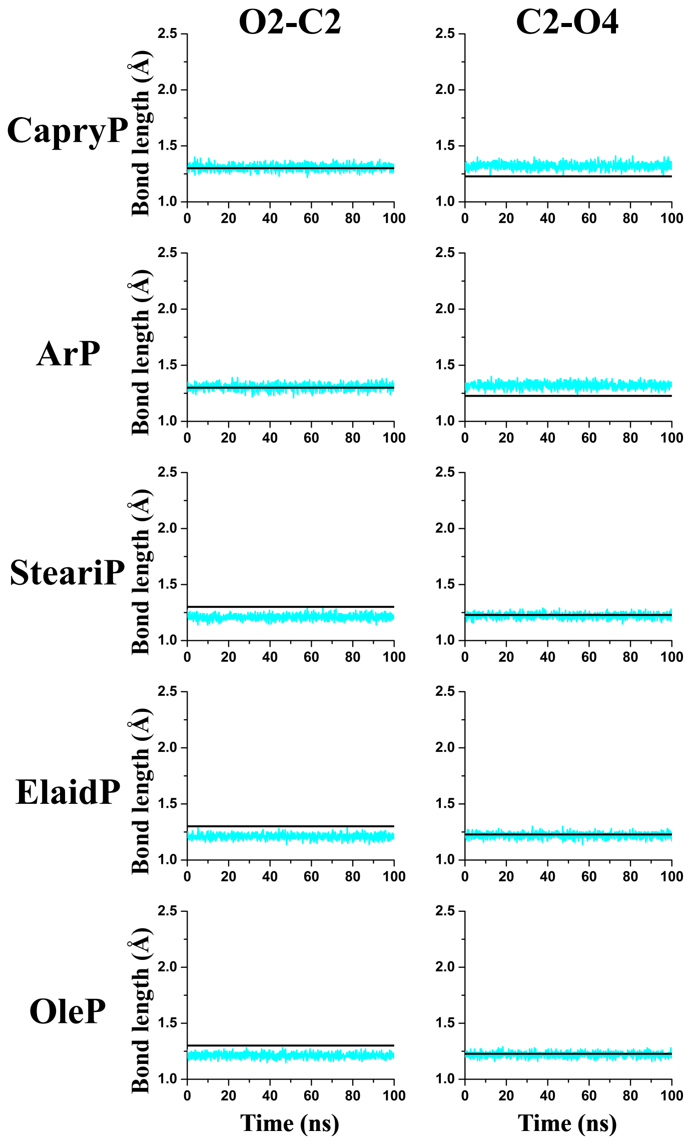

Figure S12: Time evolution of selected bonds in the Pt coordination core with the ligand over 100 ns of simulation. Shown are the axial Pt-O2 to carbonyl carbon (C2) bond and the carbonyl bond as representative examples.

Table S3: Angles (°) for the Pt coordination core obtained from (1) MD simulations, reported as average values with standard deviations in parentheses; and (2) MM energy minimization, reported as single-point values with absolute deviations from the QM reference in parentheses $\Delta = |\text{MM} - \text{QM}|$; and (3) QM optimized structures.

System	Method	N1–Pt–N2	Cl1–Pt–Cl2	N1–Pt–Cl2	N2–Pt–Cl1	O1–Pt–O2
CDDP	MD	92.94 (2.22)	94.50 (2.48)	86.24 (2.46)	86.31 (2.42)	
	MM	94.98 (3.28)	98.30 (4.12)	83.36 (3.70)	83.36 (3.70)	
	QM	91.70	94.18	87.06	87.06	
CapryP	MD	91.44 (2.14)	93.34 (2.37)	87.56 (2.30)	87.66 (2.36)	175.16 (1.68)
	MM	91.47 (1.21)	95.90 (2.62)	86.34 (1.86)	86.30 (1.97)	173.48 (0.80)
	QM	90.26	93.28	88.20	88.27	174.28
ArP	MD	91.38 (2.10)	93.23 (2.41)	87.70 (2.27)	87.70 (2.36)	175.05 (1.75)
	MM	91.47 (1.21)	95.73 (2.45)	86.50 (1.70)	86.30 (1.96)	173.50 (0.78)
	QM	90.26	93.28	88.20	88.27	174.28
SteariP	MD	91.31 (2.19)	93.24 (2.40)	87.71 (2.30)	87.75 (2.40)	174.86 (1.74)
	MM	91.88 (1.62)	95.72 (2.45)	86.24 (2.01)	86.16 (2.06)	173.51 (0.77)
	QM	90.26	93.28	88.25	88.22	174.28
ElaidP	MD	91.32 (2.18)	93.26 (2.47)	87.63 (2.32)	87.79 (2.38)	174.89 (1.74)
	MM	91.96 (1.70)	95.49 (2.27)	86.18 (2.08)	86.37 (1.88)	172.94 (1.29)
	QM	90.26	93.22	88.26	88.25	174.23
OleP	MD	91.35 (2.14)	93.42 (2.38)	87.71 (2.34)	87.53 (2.41)	174.86 (1.82)
	MM	91.87 (1.63)	95.89 (2.62)	86.08 (2.18)	86.16 (2.07)	173.54 (0.74)
	QM	90.24	93.28	88.26	88.22	174.28

References

(1) Frisch, M. J. et al. *Gaussian 16*, Revision C.01. 2016; Gaussian Inc. Wallingford CT.

(2) Yanai, T.; Tew, D. P.; Handy, N. C. A new hybrid exchange–correlation functional using the Coulomb-attenuating method (CAM-B3LYP). *Chemical physics letters* **2004**, *393*, 51–57.

(3) Dunning Jr, T. H. Gaussian basis sets for use in correlated molecular calculations. I. The atoms boron through neon and hydrogen. *The Journal of chemical physics* **1989**, *90*, 1007–1023.

(4) Kendall, R. A.; Dunning Jr, T. H.; Harrison, R. J. Electron affinities of the first-row atoms revisited. Systematic basis sets and wave functions. *The Journal of chemical physics* **1992**, *96*, 6796–6806.

(5) Figgen, D.; Peterson, K. A.; Dolg, M.; Stoll, H. Energy-consistent pseudopotentials and correlation consistent basis sets for the 5d elements Hf–Pt. *The Journal of chemical physics* **2009**, *130*.

(6) Grimme, S.; Ehrlich, S.; Goerigk, L. Effect of the damping function in dispersion corrected density functional theory. *Journal of computational chemistry* **2011**, *32*, 1456–1465.

(7) Barone, V.; Cossi, M. Quantum calculation of molecular energies and energy gradients in solution by a conductor solvent model. *The Journal of Physical Chemistry A* **1998**, *102*, 1995–2001.

(8) Seminario, J. M. Calculation of intramolecular force fields from second-derivative tensors. *International journal of quantum chemistry* **1996**, *60*, 1271–1277.

(9) Li, P.; Merz Jr, K. M. Metal ion modeling using classical mechanics. *Chemical reviews* **2017**, *117*, 1564–1686.

(10) Bayly, C. I.; Cieplak, P.; Cornell, W.; Kollman, P. A. A well-behaved electrostatic potential based method using charge restraints for deriving atomic charges: the RESP model. *The Journal of Physical Chemistry* **1993**, *97*, 10269–10280.

(11) Alviz-Amador, A.; Galindo-Murillo, R.; Pineda-Alemán, R.; Pérez-González, H.; Rodríguez-Cavallo, E.; Vivas-Reyes, R.; Méndez-Cuadro, D. Development and benchmark to obtain AMBER parameters dataset for non-standard amino acids modified with 4-hydroxy-2-nonenal. *Data in brief* **2018**, *21*, 2581–2589.

(12) Alviz-Amador, A.; Galindo-Murillo, R.; Pineda-Alemán, R.; Pérez-González, H.; Rodríguez-Cavallo, E.; Vivas-Reyes, R.; Méndez-Cuadro, D. 4-HNE carbonylation induces local conformational changes on bovine serum albumin and thioredoxin. A molecular dynamics study. *Journal of Molecular Graphics and Modelling* **2019**, *86*, 298–307.
